# Hepatocellular carcinoma: signaling pathways and therapeutic advances

**DOI:** 10.1038/s41392-024-02075-w

**Published:** 2025-02-07

**Authors:** Jiaojiao Zheng, Siying Wang, Lei Xia, Zhen Sun, Kui Ming Chan, René Bernards, Wenxin Qin, Jinhong Chen, Qiang Xia, Haojie Jin

**Affiliations:** 1 State Key Laboratory of Systems Medicine for Cancer, Shanghai Cancer Institute, Renji Hospital, Shanghai Jiao Tong University School of Medicine, Shanghai, PR China; 2 Department of Liver Surgery, Renji Hospital, Shanghai Jiao Tong University School of Medicine, Shanghai, PR China; 3 Department of Biomedical Sciences, City University of Hong Kong, Hong Kong, PR China; 4 Division of Molecular Carcinogenesis, Oncode Institute, The Netherlands Cancer Institute, Amsterdam, The Netherlands; 5 Department of General Surgery, Huashan Hospital, Fudan University, Shanghai, PR China

**Keywords:** Drug development, Translational research, Target identification

## Abstract

Liver cancer represents a major global health concern, with projections indicating that the number of new cases could surpass 1 million annually by 2025. Hepatocellular carcinoma (HCC) constitutes around 90% of liver cancer cases and is primarily linked to factors incluidng aflatoxin, hepatitis B (HBV) and C (HCV), and metabolic disorders. There are no obvious symptoms in the early stage of HCC, which often leads to delays in diagnosis. Therefore, HCC patients usually present with tumors in advanced and incurable stages. Several signaling pathways are dis-regulated in HCC and cause uncontrolled cell propagation, metastasis, and recurrence of HCC. Beyond the frequently altered and therapeutically targeted receptor tyrosine kinase (RTK) pathways in HCC, pathways involved in cell differentiation, telomere regulation, epigenetic modification and stress response also provide therapeutic potential. Investigating the key signaling pathways and their inhibitors is pivotal for achieving therapeutic advancements in the management of HCC. At present, the primary therapeutic approaches for advanced HCC are tyrosine kinase inhibitors (TKI), immune checkpoint inhibitors (ICI), and combination regimens. New trials are investigating combination therapies involving ICIs and TKIs or anti-VEGF (endothelial growth factor) therapies, as well as combinations of two immunotherapy regimens. The outcomes of these trials are expected to revolutionize HCC management across all stages. Here, we provide here a comprehensive review of cellular signaling pathways, their therapeutic potential, evidence derived from late-stage clinical trials in HCC and discuss the concepts underlying earlier clinical trials, biomarker identification, and the development of more effective therapeutics for HCC.

## Introduction

Liver cancer, primarily HCC, is among the most challenging tumors to treat, ranking sixth globally in incidence and third in cancer-related deaths.^1,2^ HBV infection is currently the leading cause of HCC, accounting for approximately 50% of all cases. However, viral infections of HBV and HCV are declining annually due to the widespread use of antiviral drugs and vaccines, while non-alcoholic steatohepatitis (NASH) linked to metabolic disorders and diabetes as a cause of HCC is on the rise.^3^

The molecular etiology of HCC differs depending on specific etiologies and genotoxic damage. The distinct molecular subtypes and immune responses elicited by HBV-associated HCC differ from those in NASH-induced HCC. A deep insight into the molecular mechanisms of HCC caused by various etiologies is required to formulate more rational therapeutic strategies. This process is complicated by the notion that only approximately 25% of HCC have actionable mutations. Nearly half of HCC patients carry at least one recurring oncogenic mutation such as TP53, CTNNB1, or TERT, However, most of these mutations lack effective targeting options with conventional pharmaceutical agents.^4^ While inhibitors designed to target mutations in the TERT promoter and components of the WNT/β-catenin signaling pathway have been developed, achieving satisfactory therapeutic effects remain elusive.^5,6^ Most other mutations remain non-targetable. Studying the effect that these mutations have on downstream signaling pathways can provide clues to the future development of drugs that target these currently undruggable mutations.

**Fig. 1:**
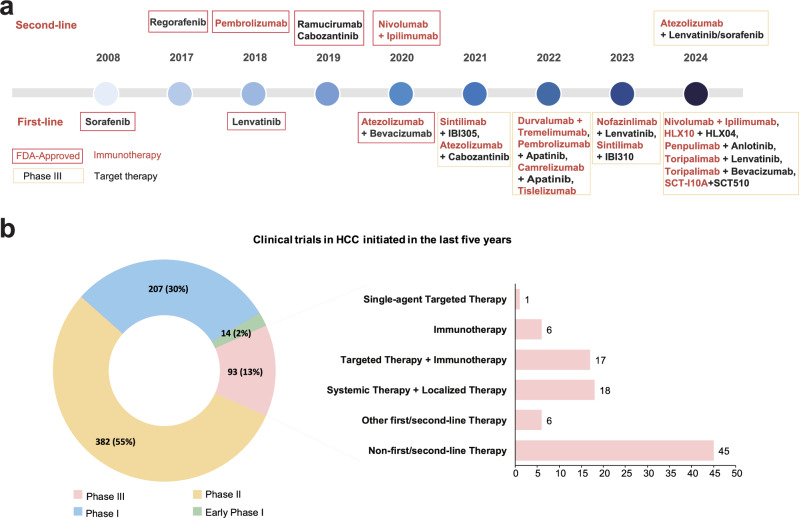
The development of targeted therapy and treatment regimens over time in HCC. **a** Describing the history of first-line and second-line therapy in HCC during 2007–2024. FDA-approved agents or agents in Phase III clinical trials framed by red and yellow colors, separately. The red represents FDA-approved drugs and black represents targeted therapy drugs. **b** Summarizing the active clinical trials over the last five years and emphasizing treatment strategies in Phase III trials. Information from the ClinicalTrials.gov registry, and those with the status of “not yet recruiting”, recruiting”, enrolling by invitation”, “active, not recruiting”, or “completed” were considered active and counted. The green color represents early Phase I, blue represents Phase I, orange represents Phase II, and red represents Phase III. Figure was created with biorender.com

The major therapeutic approaches for early stage HCC currently encompass liver resection and transplantation. In conventional approaches, local ablation utilizing radiofrequency serves as the predominant non-surgical intervention for early-stage HCC.^7^ Transarterial chemoembolization (TACE) has also been the predominant treatment method and the benchmark treatment over the past two decades for intermediate-stage HCC.^8^ Traditional methods for HCC treatment are being challenged by targeted therapy and immunotherapy (Fig. 1a). The first “targeted” therapy for advanced HCC patients is sorafenib, which gained FDA approval as a first-line therapy following successful phase III clinical trials SHARP and Asia-Pacific. The word “targeted” is between apostrophes because it is not a targeted drug in the narrow sense of the word, as it targets a number of related kinases, including RAF, MEK, VEGFR and PDGFR. The first-line treatment with sorafenib has significantly increased the median overall survival (OS), rising from 7.9 to 10.7 months. Following sorafenib’s approval, lenvatinib become another first-line agent for HCC,^9^ demonstrated prolonged anti-tumor activity, particularly in patients with portal vein invasion. The success of IMbrave150 trial in 2020 heralded a groundbreaking epoch in HCC treatment using anti PD-L1 immunotherapy (atezolizumab) synergistically intertwines with anti-angiogenic therapy (bevacizumab). This strategy showed superiority over sorafenib in terms of OS, progression-free survival (PFS), and objective response rate (ORR). Recently, the strategy also showed significantly improved recurrence-free survival (RFS) in early stage HCC.^10^ Similarly, in November 2022, FDA approved the combination of durvalumab, an anti-PD-L1 antibody, and tremelimumab, an anti-CTLA-4 (the cytotoxic T-lymphocyte-associated protein 4) antibody, which demonstrated remarkable therapeutic outcome in the HIMALAYA trial, achieving a 4-year OS rate of 25.2% for advanced HCC patients. Despite remarkable advancement, a minority of patients with advanced disease experience lasting clinical benefits, highlighting substantial therapeutic complexities.^11^

In the last five years, phase II trials constitute over half of all clinical trials in HCC (Fig. 1b). The focus of phase III clinical trials has predominantly shifted towards second-line investigations and combination therapies (Table 1), The current focus is on the development of more potent and safer agents that target immune checkpoint pathways and angiogenesis-related pathways, such as the vascular endothelial growth factor receptor (VEGFR), transforming growth factor β (TGF-β) signaling, among others. Exploring epigenetic modifications and dysregulated pathways, including MAPK, PI3K-AKT, and Janus Kinases (JAKs)/signal transducers and activators of transcription (STATs) pathway, can be beneficial in identifying drug combinations that are both safer and more effective.

**Table 1:** Phase III clinical trials of first- or second-line therapeutic strategies for hepatocellular carcinoma initiated in the last five years

Strategy	Test Drugs/Regimen	Comparator	Line	Targets	Status and results	Estimated primary completion date	Trial identifier
Targeted therapy	Namodenoson	None	Second	ADORA3	Recruiting	25-Feb	NCT05201404
Immunotherapy	AlloStim	FOLFOX regimen/ Sorafenib / Lenvatinib	First	Th1-like cells with anti-CD3/CD28 microbeads	Recruiting	1-Aug-25	NCT05033522
	Durvalumab + Tremelimumab	None	First	PD-L1; CTLA-4	Recruiting	30-Jun-25	NCT05883644
	Durvalumab + Tremelimumab	NR	First	PD-L1; CTLA-4	Recruiting	31-Mar-25	NCT05557838
	IBI310 + Sintilimab	Sorafenib	First	CTLA-4; PD-1	Recruiting	1-Dec-23	NCT04720716
	Nivolumab + Ipilimumab	Sorafenib / Lenvatinib	First	PD-1; CTLA-4	Active, not recruiting	6-Jul-26	NCT04039607
	PD-1/PD-L1 inhibitor hepatic artery infusion	PD-1/PD-L1 inhibitor vein infusion	First	PD-1/PD-L1	Recruiting	1-Jan-25	NCT03949231
Targeted therapy combined with immunotherapy	Livmoniplimab + Budigalimab	Atezolizumab + Bevacizumab / Durvalumab + Tremelimumab	First	LRRC32, TGFβ1; PD-1	Recruiting	6-Sep-30	NCT06109272
	Atezolizumab + Bevacizumab + Tiragolumab	Atezolizumab+ Bevacizumab	First	PD-L1; VEGF-A; TIGIT	Recruiting	1-Sep-26	NCT05904886
	Atezolizumab + Bevacizumab + Ipilimumab	Atezolizumab+Bevaciz umab	First	PD-L1; VEGF-A; CTLA-4	Not yet recruiting	1-Sep-24	NCT05665348
	Lenvatinib + Rulonilimab	Lenvatinib	First	VEGFR, FGFR, PDGFR, RET, KIT; PD-1	Recruiting	1-Aug-25	NCT05408221
	Atezolizumab + Bevacizumab	None	First	PD-L1; VEGF-A	Active, not recruiting	27-Apr-24	NCT04732286
	Atezolizumab + Bevacizumab	TACE	Not reported	PD-L1; VEGF-A	Recruiting	1-Apr-25	NCT04803994
	Atezolizumab + Lenvatinib / Sorafenib	Lenvatinib / Sorafenib	Second	PD-1; VEGFR, FGFR, PDGFR, RET, KIT; VEGFR, PDGFR, RAF, FLT3, KIT, FGFR, RET	Recruiting	23-Sep-24	NCT04770896
	SCT-I10A + SCT510	Sorafenib	First	VEGF; PD-1	Active, not recruiting	24-Apr	NCT04560894
	Bevacizumab + Toripalima	Sorafenib	First	VEGF-A; PD-1	Active, not recruiting	31-Oct-23	NCT04723004
	Atezolizumab + Bevacizumab	None	First	PD-L1; VEGF-A	Active, not recruiting	13-Aug-24	NCT04487067
	Lenvatinib + Toripalimab	Lenvatinib	First	VEGFR, FGFR, PDGFR, RET, KIT; PD-1	Active, not recruiting	1-May-24	NCT04523493
	Anlotinib + AK105	Sorafenib	Not reported	VEGFR, PDGFR, FGFR, KIT; PD-1	Not yet recruiting	30-Jun-24	NCT04344158
	Lenvatinib + Nofazinlimab	Lenvatinib	First	VEGFR, FGFR, PDGFR, RET, KIT; PD-1	Active, not recruiting	30-Jun-25	NCT04194775
	Apatinib + Camrelizumab	Sorafenib	First	VEGFR; PD-1	Completed	8-Feb-22	NCT03764293
	Lenvatinib + Pembrolizumab	Lenvatinib	First	VEGFR, FGFR, PDGFR, RET, KIT; PD-1	Active, not recruiting	21-Jun-22	NCT03713593
	Cabozantinib + Atezolizumab	Cabozantinib / Sorafenib	First	MET, VEGFR, ROS1, RET, AXL, NTRK, KIT; PD-L1	Active, not recruiting	8-Mar-21	NCT03755791
	Atezolizumab + Bevacizumab	Sorafenib	First	PD-L1; VEGF-A	Completed	31-Aug-20	NCT03434379
Localized TACE/HAIC therapy combined with systemic therapy	HAIC-FOLFOX + Lenvatinib + Toripalimab	HAIC-FOLFOX + Lenvatinib	First	VEGFR, FGFR, PDGFR, RET, KIT; PD-1	Recruiting	26-Jul-25	NCT06201065
	TACE + Bevacizumab + Sintilimab	TACE + Lenvatinib	First	VEGF-A; PD-1	Recruiting	31-Jul-27	NCT05985798
	HAIC-FOLFOX + Apatinib + Camrelizumab	Intravenous FOLFOX + Camrelizumab + Apatinib	First	VEGFR; PD-1	Recruiting	31-Jul-26	NCT06172205
	HAIC-FOLFOX + Zoledronic acid	HAIC-FOLFOX	First	Farnesyl pyrophosphate synthase, hydroxylapatite, geranylgeranyl pyrophosphate synthase	Recruiting	1-Dec-24	NCT05866172
	TACE + SBRT + Lenvatinib	Lenvatinib	First	VEGFR, FGFR, PDGFR, RET, KIT	Not yet recruiting	27-Feb	NCT05718232
	TACE + Lenvatinib + Sintilimab	TACE + Lenvatinib	First	VEGFR, FGFR, PDGFR, RET, KIT; PD-1	Recruiting	31-Oct-26	NCT05608200
	TACE + Anlotinib + Penpulimab	Penpulimab + Anlotinib	Not reported	VEGFR, PDGFR, FGFR, c-Kit; PD-1	Recruiting	31-Mar-23	NCT05344924
	TACE + Apatinibmesylate + Camrelizumab	TACE	Not reported	VEGFR; PD-1	Recruiting	30-Jul-24	NCT05320692
	HAIC-FOLFOX + Apatinib + Camrelizumab	Apatinib + Camrelizumab	First	VEGFR2; PD-1	Recruiting	1-Nov-24	NCT05313282
	TACE + synchronous Lenvatinib	TACE + sequential Lenvatinib	First	VEGFR, FGFR, PDGFR, RET, KIT	Recruiting	28-Feb-24	NCT05220020
	TACE + Tremelimumab + Durvalumab + (Lenvatinib)	TACE	Not reported	CTLA-4; PD-L1; VEGFR, FGFR, PDGFR, RET, KIT	Recruiting	31-Dec-25	NCT05301842
	HAIC-FOLFOX + Apatinib + Camrelizumab	Apatinib + Camrelizumab	First	VEGFR2; PD-1	Recruiting	1-Jan-26	NCT05198609
	TACE + Atezolizumab + Becavizumab	TACE	First	PD-L1; VEGF-A	Active, not recruiting	28-Feb-29	NCT04712643
	TACE + Lenvatinib + Pembrolizumab	TACE	Not reported	VEGFR, FGFR, PDGFR, RET, KIT; PD-1	Active, not recruiting	30-Jun-28	NCT04246177
	TACE + SBRT + Sorafenib	Sorafenib	First	VEGFR, PDGFR, RAF, FLT3, KIT, FGFR, RET	Recruiting	1-Aug-23	NCT04387695
	TACE/TAE + Nivolumab	TACE/TAE	First	PD-1	Recruiting	25-Jun	NCT04268888
	TACE + Bevacizumab + Durvalumab / TACE + Durvalumab	TACE	Not reported	VEGF-A; PD-L1	Active, not recruiting	11-Sep-23	NCT03778957
	HAIC-FOLFOX-5 FU + Concurrent Lenvatinib+ Concurrent PD-1 antibody	HAIC-FOLFOX-5 FU + Sequential Lenvatinib + Sequential PD-1 antibody	First	VEGFR, FGFR, PDGFR, RET, KIT; PD-1	Recruiting	27-Jul	NCT06041477
Other strategies	QL1706 + Bevacizumab + chemotherapy (Oxaliplatin + Capecitabine) / QL1706 + Bevacizumab / QL1706 + chemotherapy	Sintilimab + Bevacizumab	First	PD-1; CTLA-4; VEGF-A	Not yet recruiting	1-Sep-27	NCT05976568
	Icaritin	Huachansu	First	NR (Traditional Chinese Medicine)	Recruiting	30-Aug-25	NCT05594927
	Lenvatinib + I-125 Seed Brachytherapy	Lenvatinib	First	VEGFR, FGFR, PDGFR, RET, KIT	Recruiting	31-Oct-26	NCT05608213
	ADI-PEG20	Placebo	Second	Pegargiminase	Recruiting	25-Sep	NCT05317819
	Hepatectomy + Camrelizumab + Apatinib	None	Not reported	PD-1; VEGFR	Recruiting	30-Sep-24	NCT05062837
	Radiotherapy + Toripalimab	Sorafenib	Not reported	PD-1	Recruiting	31-Mar-24	NCT04709380

Information retrieved from “https://classic.clinicaltrials.gov/”, as of April 15, 2024

This review summarizes essential signaling pathways including cellular signaling pathways and immune-related signaling pathways, discusses current potential therapeutic targets in HCC, and presents preclinical animal models alongside ongoing or completed clinical studies on systemic therapy, aiming to provide a prospective outlook on precision treatment for HCC in the future.

## Cellular signaling pathways in HCC cells

Cellular signaling pathways in HCC cells typically bifurcate into two main segments: the RTK signaling pathways and other pathways. Examining the application of these two kinds of pathways in preclinical and clinical contexts reveals their pivotal roles in processes such as cell proliferation, survival and migration. Understanding these pathways holds promise for devising targeted therapies and advancing cancer treatment strategies.

### RTKs signaling pathways

The RTKs comprise a cluster of transmembrane receptors that are activated upon binding to specific ligands, such as growth factors or hormones.^12^ The RTKs consist of an extracellular domain for ligand binding, transmembrane regions, and a cytoplasmic domain with tyrosine kinase activity and an ATP-binding site.^13^ These structural components allow RTKs to transduce extracellular signals into intracellular responses by activating downstream signaling pathways. Upon ligand binding, RTKs undergo dimerization, leading to autophosphorylation of tyrosine residues within their cytoplasmic domains. This autophosphorylation activates the intrinsic kinase activity of the receptor, allowing it to phosphorylate tyrosine residues on downstream signaling molecules. These phosphorylation events initiate intracellular signaling cascades, such as MAPK (mitogen-activated protein kinase), that are essential for cell survival, proliferation, and differentiation. Dysregulation of RTK signaling pathways is associated with HCC, making them important therapeutic targets. Based on the accumulated knowledge from existing studies, we focused on reviewing and discussing the roles of eight RTKs classes including VEGF receptor (VEGFR), Epidermal growth factor receptor (EGFR), c-Met, platelet-derived growth factor (PDGFR), fibroblast growth factor receptor (FGFR), insulin-like growth factor receptor (IGFR), KIT, RET (Rearranged during transfection) and their downstream pathways including MAPK pathway, Phosphatidylinositol 3-kinase (PI3K) pathway and JAK-STAT pathway in biological behaviors of HCC as well as their therapeutic implications.

### VEGFR

“Sustained angiogenesis” is identified as one of the hallmarks of cancer proposed by Drs. Weinberg and Hanahan as early as 2000.^14^ Malignant tumor cells require sufficient nutrients and oxygen for rapid survival and proliferation; thus, tumor neovascularization is crucial in supporting tumor growth, infiltration, and spread.^15,16^ HCC is a vascular-rich solid tumor with hypervascular nature. Neovascularization plays an important role in the development of HCC, and therefore most of the existing targeted therapies are aimed at targeting its angiogenic pathways.^17,18^ VEGF/VEGFR pathway is one of the most typical and prominent tumor-induced pro-angiogenic factors affecting the development of HCC.^19^ VEGFA has a 7% ~ 14% frequency of focal amplification in HCC.^20,21^ Its receptors, VEGFR-1 and VEGFR-2, are usually highly expressed in HCC and correlated with the differentiation and stage of the tumors.^22–25^ In HCC patients, circulating VEGF serves as a marker for HCC metastasis, whilst VEGF signaling maintains an immunosuppressive tumor microenvironment.^26^

Almost all approved TKI for advanced HCC involve the targets of VEGFRs. Cancer neoangiogenesis is mainly mediated through the VEGFA/VEGFR2 axis, making VEGFA and VEGFR2 key targets for the development of novel drugs. Inhibition of VEGFR signaling has shown promising antitumor effects in HCC cell lines and mouse models.^27,28^ Furthermore, a number of selective VEGFR-related inhibitors are already in clinical practice. Bevacizumab, a recombinant humanized monoclonal antibody against VEGFA, was the first FDA-approved agent to inhibit angiogenesis and has proven to be effective against a wide range of advanced malignancies.^29^ The combination of bevacizumab and PD-L1 monoclonal antibody atezolizumab was authorized as a first-line therapy for advanced HCC in 2020.^30^ Compared with sorafenib alone, atezolizumab plus bevacizumab significantly prolonged overall and disease-free survival and maintained clinical efficacy during long-term follow-up.^30,31^ This combination has plausible biological mechanisms that restore or enhance the efficacy of immune checkpoint inhibitors in antitumor therapy by inhibiting the VEGFR-associated immunosuppressive microenvironment.^32^ Several other regimens targeting VEGFR signaling in combination with PD-1/-L1 inhibitors have now entered phase III clinical trials (NCT06172205, NCT05320692, NCT03764293). The CARES-310 study (NCT03764293), which recently disclosed data, demonstrated that apatinib, a selective VEGFR2 small molecule inhibitor, in combination with the PD-1 monoclonal antibody camrelizumab had a superior benefit over sorafenib, and therefore this combination is currently approved by the National Medical Products Administration (NMPA) as first-line therapy for unresectable or metastatic HCC.^33^ Moreover, apatinib monotherapy, which prolonged survival and response rates in advanced HCC compared to placebo as a second-line therapy, has now been approved by the NMPA. Following this, another VEGFR2 monoclonal antibody, ramucirumab, has been approved by the FDA for advanced HCC patients who previously recieved sorafenib and had an AFP ≥ 400 ng/mL.^34^ The success of these agents demonstrates the substantial role of VEGFR in HCC treatment. But there have been some failures, for example, the dual VEGFR and FGFR inhibitor brivanib failed in phase III trials as first-line, second-line, and adjuvant therapy in HCC.^35–37^ Two pan-VEGFR inhibitors, axitinib and cediranib, failed to advance to phase III trials after poor performance in phase II trials.^38,39^ These failures may be attributed in part to flaws in study design, such as imbalance in patient baseline across treatment arms and lack of specific participant eligibility criteria, but may also be due to the structure of the agents themselves being less active in humans and presenting off-target toxicity.

### EGFR

EGFR belongs to the ErbB receptor family, which is expressed in many organs of the human body at low levels. Compared with other tissues, EGFR is relatively highly expressed in hepatocytes of the adult liver, indicating its significance in maintaining liver function.^40^ As a potential biomarker of drug resistance in tumors, EGFR exhibits intricate responses to various cellular stresses, including drug stress, UV radiation, and inflammatory cytokines. Mutations in EGFR can occur in various solid malignant tumors, especially in Asian NSCLC patients.^41,42^ Previous studies reported that activating mutations in exons 18-21 of EGFR are rare in HCC patients.^43,44^ Nevertheless, one study found 13 novel missense mutations in EGFR exon 19-23 from HCC tissues,^43^ and seven of these HCC-derived mutants (K757E, N808S, R831C, V897A, P937L, T940A, and M947T) showed resistant to first-generation EGFR inhibitors.^45^ For these patients with rare EGFR mutations, gefitinib is not suitable for combination with lenvatinib but later-generation EGFR inhibitors, such as osimertinib, could be considered.

EGFR is expressed in over 60% of HCC patients, and is positively correlated with poor differentiation, high proliferative activity, advanced stage, intrahepatic metastasis, and poorer PFS.^46–49^ EGFR exhibits high expression levels in HCC cell lines and tissues, and furthermore, up-regulated EGFR expression in circulating tumor microemboli maintains stability and distal metastasis of suspended HCC cells by activating Ras/MAPK pathway.^50^ The high EGFR-related pathway signaling in HCC may be related to alterations in its regulatory factors and overexpressed ligands.^20,51,52^

EGFR signaling has been found to mediate resistance to the first-line targeted drugs lenvatinib and sorafenib. Our group previously identified EGFR as a synthetic lethal target of lenvatinib in HCC by using CRISPR-Cas9 genetic screen.^53^ One of the mechanisms of intrinsic resistance to lenvatinib treatment in HCC is the inhibition of fibroblast growth factor receptor (FGFR) leading to a feedback activation of the EGFR-PAK2-ERK1/2 and EGFR-PAK2-ERK5 signaling pathways, which allows malignant cells to maintain survival and sustain proliferation. Subsequent phase I clinical trials demonstrated a strong synergistic effect of lenvatinib in combination with the EGFR inhibitor gefitinib in advanced HCC with high expression of EGFR and refractory to lenvatinib mono-therapy, with 9 out of 30 cases achieving partial response (NCT04642547). Recent studies have also revealed multiple other mechanisms of EGFR involved in lenvatinib resistance, including reactive oxygen species accumulation,^54^ activation of ABCB1 to potentiate the cytotoxic effects of lenvatinib,^55^ and as a downstream target for RNA modification.^56,57^ An analysis of circulating tumor DNA profiling of peripheral blood suggests that pre-treatment low copy number EGFR/ERBB2 amplification may serve as a genetic marker for lenvatinib resistance.^58^ Moreover, many studies have shown that EGFR also contributes to primary and acquired resistance to sorafenib in HCC cells and can serve as a potential predictor of resistance to sorafenib.^59–62^ However, our preclinical data demonstrated that sorafenib in combination with EGFR inhibitors did not show a synergistic effect in the majority of liver cancer cell lines,^53^ and the phase III RCT trial also found that the addition of erlotinib, a selective inhibitor targeting EGFR tyrosine kinase, to sorafenib exhibited no enhancement in survival outcomes for advanced HCC.^63^

### C-Met

The c-Met is expressed primarily on epithelial cells and is a transmembrane receptor containing the catalytic structural domain of tyrosine kinase. The only known endogenous ligand is HGF, which is secreted mainly by mesenchymal cells and is a hepatocyte mitogen.^64^ HGF can activate downstream MAPK, PI3K/AKT, and STAT3 signaling pathways upon binding to c-Met via low (the SPH domain) or high (the N and kringle 1 domains) affinity binding sites. It is involved in various physiological processes such as embryonic development, tissue repair and regeneration, and inflammatory responses.^65^ Furthermore, it also transmits signals in a noncanonical manner independent of HGF, which is an important mechanism for malignant cells to acquire resistance to inhibitors in pathological conditions.^66–68^ MET is considered a proto-oncogene, and together with its ligand HGF have been found to be dysregulated in the process of HCC development.^69^ Downstream signaling pathways activated by c-Met contribute to malignant actions including survival, growth, invasion, metastasis, metabolic reprogramming, epithelial-mesenchymal transition (EMT), drug resistance, and enhancement of tumor stemness.^65^ Although c-Met mutation or amplification is detected in only approximately 1% of HCC patients,^70^ its overexpression occurs in about a quarter of patients, even up to 80% in advanced HCC.^71,72^ Activation of HGF/c-Met is one of the key mechanisms of resistance to sorafenib and lenvatinib, which is associated with the bypassing of growth factor targets of sorafenib and lenvatinib by HGF/c-Met to promote the downstream pro-carcinogenic MAPK pathway and induction of EMT.^73^ Therefore, c-Met levels could serve as a potential biomarker for predicting response to sorafenib and lenvatinib.^24,74–77^ Sorafenib or lenvatinib in combination with c-Met inhibitors exhibited therapeutic sensitization and synergistic antitumor effects.^74,76^ However, the prognostic association between HGF/c-Met and HCC patients is still uncertain; although most studies have shown that high c-Met or HGF expression in HCC predicts shorter survival and higher potential for recurrence and metastasis,^78–81^ some studies have also suggested no prognostic significance of c-Met and HGF.^71,78^

Several inhibitors targeting HGF/c-Met have been developed. Cabozantinib is a c-Met/RTK inhibitor, which is currently being used for the treatment of HCC patients as a second-line drug. The recent results of the phase III trial COSMIC-312 demonstrated that cabozantinib in combination with the PD-L1 inhibitor atezolizumab prolonged PFS compared to sorafenib, but did not improve OS.^82^ Some selective c-Met inhibitors have been tested for antitumor activity in HCC, but blocking c-Met alone does not appear to provide an adequate response.^83^ Tivanitib, an oral selective c-Met inhibitor, showed encouraging responses in preclinical studies and a phase II clinical trial, but a subsequent phase III trial in advanced HCC with high c-Met expression did not demonstrate similar effects.^84,85^ Survival was not improved with tivanitib monotherapy compared with the placebo group. The failure may be attributable to primary resistance to c-Met inhibitors and an exaggerated role for highly expressed c-Met in patients previously treated with sorafenib.^86^

Given the contribution of c-Met to sorafenib and lenvatinib resistance as demonstrated by preclinical data, a strategy of tivanitib in combination with sorafenib and lenvatinib has been proposed,^76,77,87^ but remains to be validated in clinical settings. Interestingly, two highly selective c-Met inhibitors that have been approved by the FDA for the indication of NSCLC harboring the METex14 skipping mutation, tepotinib and capmatinib, have shown promising efficacy in early clinical trials in HCC.^88–90^

### FGFR

The FGFR gene family, including FGFR1-4, encodes seven FGFR proteins, since FGFR1-3 have two alternative splice variants.^91^ Eighteen ligands and four homologs have been identified in the fibroblast growth factor (FGF) family.^92^ Originally named for their ability to promote mitogenesis in fibroblasts, FGFs are now recognized as one of the most diverse families of peptide growth factors in mammals. A substantial amount of evidence has shown dysregulation of FGFR signaling as a pathogenic mechanism and consequence of cancer,^93^ and it is frequently detected to be highly expressed in HCC, contributing to tumorigenesis, progression, and drug resistance.^94,95^ Despite the relatively low frequency of genetic alterations, dysregulated FGF/FGFR signaling affects about half of HCC patients.^96–98^ Among the FGF and FGFR families, currently the most studied and promising target in HCC is the FGF19-FGFR4 signaling pathway. FGF19 is localized in the 11q13.3 amplicon, and is frequently detected co-amplified with cyclin D1 (CCND1), a known proto-oncogene, resulting in increased expression.^98^ They have been found to be key oncogenic driver signals in HCC and are widely expressed in patients promoting HCC progression.^98,99^ Transgenic mice overexpressing FGF19 in skeletal muscle led to the development of HCC,^100^ which was effectively inhibited by the use of either anti-FGF19 or FGFR4 monoclonal antibodies.^101,102^ Interestingly, clinical evidence showed that the expression of FGF19 and FGFR4 increased sequentially with the different histologic stages of carcinogenesis (steatosis-steatohepatitis–cirrhosis-HCC), suggesting a strong association with HCC development.^103,104^ In addition, FGF19 can exert pro-oncogenic effects by crosstalk activation of other growth factor pathways, such as increasing the expression of the EGFR ligands, connective tissue growth factor (CTGF) and amphiregulin, thereby affecting the development of HCC through indirectly or directly shared pathways.^105,106^ Furthermore, FGF19/FGFR4 confers acquired resistance to multikinase inhibitor therapy in HCC, and targeting the FGF19/FGFR4 axis can sensitize the antitumor effects of sorafenib and regorafenib.^94,107,108^

FGFR4 is specifically expressed in the liver, making it a promising target for drug development. Moreover, the hinge region of the FGFR4 complex has a unique non-conserved Cys552 structure, whereas the corresponding positions of FGFR1-3 are tyrosine, providing feasibility for the development of selective covalent FGFR4 inhibitors.^109^ Pan-FGFR inhibitors that have been approved by the FDA are erdafitinib, pemigatinib, and infigratinib for indications including uroepithelial cancers, bile duct cancers, or myeloid/lymphoid neoplasms.^110,111^ Compared to these pan-FGFR inhibitors, selective FGFR4 inhibitors have better manageable toxicity. FGFR4 inhibitors that have shown promising anti-cancer activity in HCC include BLU-9931,^112,113^ H3B-6527,^114^ FGF-401,^115^ CXF-007,^116^ and BLU-554 ^117^. Currently, phase I or phase II clinical trials have been completed for H3B-6527, BLU-554, and FGF-401, all of which have shown good tolerability.^118^ The first-line RTK inhibitor lenvatinib also can suppress FGFRs, but, the degree of its FGFR4 inhibition remains uncertain.

### IGFR

The IGFR consist of two isoforms, IGF-1R and IGF-2R, which together with the ligands IGF-1 and IGF-2, and the high affinity IGF-binding protein 1-6 (IGFBP 1-6), comprise the IGF family. IGF-1 exerts its biological effects by binding to IGF-1R, whereas IGF-2 binds to IGF-1R, IGF-2R, and insulin receptor.^119^ Both have prominent roles in the growth and development of the organism, and their deficiencies lead to developmental disorders.^120^ Due to shared receptors and ligands, the IGF-1R signaling cascade is also crosstalked by the insulin/insulin receptor signaling pathway.^121^ In contrast, IGF-2R is a type I transmembrane protein with no kinase activity and does not transduce signaling. It competitively binds to IGF-2 and enters the lysosome via endocytosis for degradation to impair IGF-IR signaling.^122^ The association between the IGF family and HCC development has been widely reported. Overexpressed IGF-2 and IGF-1R are frequently detected in HCC tissues and correlate with advanced stage and prognosis of HCC patients.^123–126^ Interestingly, although IGF-IR has no nuclear localization sequence and is normally localized to the cytoplasmic membrane, it can undergo nuclear ectoposition by SUMOylation, binding to promoter DNA of its own gene and some oncogene-related genes, such as JUN and FAM21, to promote gene expression.^123,127,128^ IGF-2R is considered to harbor tumor suppressor gene properties due to inhibition of IGF-1R signaling caused by competitive binding of IGF2. Besides antagonizing IGF-1R, IGF-2R promotes the transport of lysosomal enzymes and the tumor suppressor TGF-β1 into cells to exert inhibitory effects on tumor development.^122,129^ Thus, in contrast to the overexpression of IGF-1R in patients, the locus where the IGF-2R gene resides has been found to have frequent loss of heterozygosity (LOH) and reduced expression in different cancers, especially HCC.^130–132^

The IGF axis, due to its high relevance with tumorigenesis and development in HCC, has emerged as a viable target for therapeutic intervention. Considerable efforts were made to block the IGF-1R signaling pathway at multiple levels. To date, three primary classes of therapeutic agents have been assessed in both animal models and clinical trials: small molecular tyrosine kinase inhibitors, antibodies to IGF-1R, and antibodies to IGF ligands. Additionally, alternative therapeutic strategies, including small interfering RNAs, antisense oligonucleotides, recombinant IGFBPs, and pregnancy-associated plasma protein-A (PAPP-A) inhibitors, have also been explored.^119^ The only currently approved selective IGF-1R inhibitor is teprotumumab, a fully humanized IGF-1R monoclonal antibody, which is used to treat thyroid eye disease.^133^ Decades of effort have gone into the development of IGF axis-targeted drugs for indications in oncology. Unfortunately, although some therapies targeting the IGF axis have demonstrated potent antitumor activity and tolerability in preclinical and early clinical trials, no drug has successfully passed phase III trials to date.^134,135^ The response to targeting IGF1R as monotherapy is limited in most cases,^136^ and therefore it is often used in combination with other targeted therapies or chemotherapy, but the results remain disappointing. For patients with HCC, the IGF-1R inhibitor cixutumumab tested in the clinical setting showed limited clinical benefit.^137,138^ Failure is often attributed to the activation of compensatory signaling pathways, such as growth hormone- or insulin-related pathways, in response to IGF axis inhibition, resulting in the loss of inhibition of downstream MAPK and PI3K-AKT-mTOR cascades.^138–140^ In addition, crosstalk between IGF-1R and other receptors such as insulin receptor, EGFR, and integrins increases the complexity of IGF axis targeting.^138,141,142^ Therefore, further insight into the complex signaling network between the IGF axis and other pro-cancer pathways and the identification of biomarkers that predict therapeutic response are important and urgent.

### PDGF

The PDGF family encoded by four genes, PDGFA, PDGFB, PDGFC, and PDGFD.^143,144^ The PDGF receptor (PDGFR) contains two isoforms, PDGFR-α and PDGFR-β, and is a type III tyrosine kinase protein.^145–147^ PDGFs are typically produced by discrete cell populations and act as paracrine factors to regulate cellular responses.^148,149^ PDGFRs are considered as an oncogenic driver, expressed or subject to activating genetic alterations in many types of cancer tissues, including HCC, which promote carcinogenesis, progression, and drug resistance through the classical MAPK and PI3K cascades.^150–152^ More than half of HCC patients have high PDGFR-α expression in their tumor tissues.^153^ Overexpressed PDGFRs promote HCC progression and are significantly associated with a worse prognosis of HCC patients.^24,154^ Similar to the functions of VEGF and FGF, PDGFs also can act as a pro-angiogenic factor in HCC tissues and has a role in inducing tumor neovascularization ^17^. Upregulated PDGFR-α may be involved in HCC development and is significantly associated with microvessel density and vascular infiltration of tumors,^154^ which could serve as a biomarker for predicting HCC metastasis and a potential therapeutic target.^155^

Several currently FDA-approved HCC-targeted agents, including lenvatinib, sorafenib, and regorafenib, have PDGFR as one of their targets. Donafenib monotherapy, which simultaneously targets PDGFR, VEGFR, and RAF kinases, was approved by the NMPA in 2021 as a first-line therapy for advanced HCC based on its ability to achieve a superior OS compared with sorafenib.^156^ Linifanib, a small molecule inhibitor targeting PDFGR and VEGFR, was found to have promising antitumor activity in HCC in preclinical and early clinical studies.^157^ However, it did not show superior efficacy over sorafenib in phase III trials and failed to meet the primary endpoint.^158^ Another triple angiokinase inhibitor, nintedanib, targeting PDFGR/VEGFR/FGFR, also exhibited similar results in clinical trials.^159^

Approaches to selectively inhibiting the PDGF/PDGFR axis primarily include receptor antibodies or small molecule inhibitors that block ligand-receptor interactions, neutralizing antibodies or ligand traps that isolate the ligand, and blocking PDGFR kinase domain activity.^152^ However, there are several challenges to specifically targeting PDGFR signaling. PDGFR-α and PDGFR-β, as class III RTK proteins, have greater structural similarity to some other cell surface receptors such as FLT3, kit, and CSF1R, as well as functional redundancy when PDGFR is activated.^152,160^ Several selective PDGFR inhibitors have been developed, including olaratumab, CP-673451, and CHMFL-PDGFRα-159, all of which have shown significant anti-cancer and antiangiogenic effects.^161–165^ One of these, olaratumab, was tested clinically and was received accelerated approval by the FDA for the treatment of advanced soft tissue sarcoma based on a successful phase II result, but unfortunately did not show similar benefit in a subsequent phase III trial.^166^ In addition, anti-PDGFR-α monoclonal antibodies have been found to have anti-proliferative and pro-apoptotic effects in multiple human and mouse HCC cell lines.^153^ Inhibition of PDGF signaling to suppress HCC progression was also found in mouse models.^167^ However, there are currently no selective PDGF/PDGFR inhibitors in clinical testing for HCC.

### C-Kit

c-Kit, encoded by the KIT gene, is primarily activated by stem cell factor (SCF).^168^ In normal conditions, c-Kit is abundantly expressed in hematopoietic stem cells and is crucial for cell survival, proliferation, and differentiation.^169^ The current view is that c-Kit may play a double-edged role in the liver: on the one hand, c-Kit-positive cells participate in tissue repair by promoting target cell regeneration in the event of liver injury, but on the other hand, the overexpression or mutation of c-Kit as a proto-oncogene contributes to the development of HCC.^170^ An important contributor to heterogeneity in HCC is liver cancer stem/progenitor cells (LCSCs), implicated in drug resistance and tumor recurrence.^171^ c-Kit receptor serves as a marker for LCSCs, and its overexpression promotes the transformation of hepatic stem/progenitor cells into LCSCs.^172–174^ In HCC driven by HBV or HCV, c-Kit plays a mediating role. PreS1 protein of HBV and core proteins of HCV induce LCSC production and self-renewal of tumor cells by stimulating c-Kit expression, accelerating HCC onset and progression.^175–177^ Aberrant crosstalk exists between SCF/c-Kit and other pro-cancer pathways like TGF-β/SMAD2, forming positive feedback loops promoting malignant cell proliferation and invasion.^178^

The first targeted drug approved for the treatment of cancer, imatinib, has c-kit as one of its targets. Unfortunately, clinical trials of imatinib in HCC have all failed.^179,180^ Many inhibitors targeting c-Kit mutations have been approved, but the vast majority are multi-targeted kinase inhibitors.^181^ One of the targets of the current first-line targeted agents sorafenib and lenvatinib and the second-line therapies regorafenib and cabozantinib in the treatment of HCC includes c-Kit. Another tyrosine kinase inhibitor, anlotinib, which takes c-Kit as one of its targets, has shown promising efficacy and a tolerable safety profile in clinical studies. In a multicenter, phase II study enrolling patients with advanced HCC, anlotinib in combination with the PD-1 inhibitor toripalimab as first-line treatment achieved an ORR of 32% and a median survival of 18.2 months.^182^ Another phase II studies found that anlotinib monotherapy resulted in promising benefits as first- or second-line treatment in advanced HCC,^183^ while the combination of chemotherapy did not provide additional efficacy.^184^ Several real-world studies have also confirmed the efficacy of anlotinib in patients with HCC.^185,186^ Multiple Phase III clinical trials are currently testing the efficacy of anlotinib in HCC patients in different clinical settings (NCT05862337, NCT04344158, NCT05344924, NCT04665609, NCT03950518). However, anlotinib also targets some other RTKs, and the effect of selective c-Kit inhibitors on HCC has been less well-studied.

### RET

RET is identified in 1985 during the transfection of mouse NIH-3T3 cells with human T-cell lymphoma.^187^ In the normal body, RET has a crucial role in embryonic kidney and neural development.^188,189^ RET is considered a proto-oncogenic driver gene, and RET fusion is the alteration of great interest, whereby the RET gene breaks and recombines with another gene to form a new gene, thus possessing the ability to self-phosphorylate for sustained activation, frequently occurring in thyroid cancer and NSCLC.^190^ Treatment of EGFR or KRAS inhibitors may induce acquired RET fusions, contributing to resistance to targeted therapy.^191,192^ RET alterations are rare in HCC patients with a frequency of less than 1%.^193,194^ Data from TCGA suggest that RET expression is significantly downregulated in HCC tissues compared to adjacent tissues.^195^ Currently, the mechanism of RET signaling in the occurrence and development of HCC has been less reported.

The first-line drug lenvatinib and the second-line drugs cabozantinib and regorafenib, used for advanced HCC treatment, target RET among other pathways. FDA-approved selective RET inhibitors, pralsetinib and selpercatinib, are indicated for metastatic RET fusion-positive NSCLC and medullary thyroid cancer, with selpercatinib additionally approved for other advanced RET fusion-positive solid tumors.^196,197^ However, the multicancer indication for selpercatinib was based on a phase I/II trial excluding HCC patients.^190^ Although lacking preclinical and clinical data in HCC, the success in thyroid and lung cancers prompts exploration of RET inhibitor efficacy in HCC, necessitating patient selection criteria.

### MAPK pathway

The MAPK pathway is one of the most important signaling pathways underlying life-sustaining activities in eukaryotes, which is frequently altered in a wide range of diseases.^198^ Four different MAPK cascades have been identified, namely the extracellular signal regulated kinase 1/2 (ERK1/2), Jun amino terminal kinases (JNK), p38 MAPK, and BMK1 cascades. ERK1/2 is the most classical and well-studied key MAPK signaling pathway, which is activated mainly by signals transmitted through cell surface transmembrane receptors such as RTKs or G protein-coupled receptors.^198,199^ Almost all growth factor signals depend on the activation of ERK to complete the signaling process, thus ERK is a ubiquitous signaling pathway in the human body. Ras, Raf, MEK and ERK proteins are key components of this pathway, and abnormal function of any one of them may lead to serious consequences. Since ERK plays an important role in key cellular functions, its aberrant activation is closely linked to the development of malignant lesions. The upstream activating protein of the ERK pathway, RAS is a frequent driver mutation in human cancers, with approximately 19% of cancer patients harboring mutations in the RAS gene.^200,201^ Aberrant activation of the MAPK pathway is present in about half of patients with early-stage HCC, and high expression of ERK signaling is detected in almost all patients at advanced stages.^202–204^ Activated MAPK signaling is significantly associated with poorer prognosis and metastasis in HCC patients.^205,206^

Inhibitors targeting the MAPK pathway are a hot topic in antitumor drug development and have great potential for clinical application. Almost all of the currently approved RTK-targeted agents in the HCC could affect downstream activation of the MAPK pathway. One of the targets of both sorafenib and regorafenib includes the RAF protein. A number of selective inhibitors have been developed that target components of the MAPK pathway, focusing on two key kinases, MEK and ERK. The first MEK inhibitor to enter clinical testing was CI-1040 (also known as PD184352), which targets MEK1/2. Although CI-1040 in combination with sorafenib was found to have a superior antitumor effect compared to sorafenib alone in a xenograft HCC model,^207^ it displayed insufficient antitumor activity in a phase 2 clinical study in solid tumors,^208^ which may be due to its low exposure resulting from its fast clearance rate and poor solubility. Several second-generation selective MEK inhibitors have subsequently been developed, which has led to the approval of the selective MEK1/2 inhibitors trametinib, cobimetinib, selumetinib, and binimetinib. In addition, three RAF inhibitors, vemurafenib, dabrafenib, encorafenib, and two KRAS^G12C^ inhibitors, sotorasib and adagrasib, have also become available in recent years.^209^ Although none of their current indications include HCC, promising antitumor activity in HCC has been demonstrated in several preclinical and early clinical trials, either as a monotherapy or in conjunction with other targeted agents.^210–214^ For instance, a phase II clinical trial found that the MEK1/2 inhibitor refametinib in combination with sorafenib demonstrated potential synergistic effects in HCC patients with Ras mutations.^215^ Although therapies targeting the MAPK pathway have made encouraging progress, they are highly susceptible to drug resistance. The therapeutic effect is often short-lived and difficult to achieve complete tumor regression.^216^ Activating mutations of key components under drug stress, mobilization of independent alternative pathways, and feedback upregulation of the number of targeted proteins can all lead to resistance to MAPK pathway-related inhibitors.^217^ Several coping strategies have been proposed, including rational combinations (such as MEK plus BRAF inhibitors) and optimization of the drug’s structure (such as the second-generation RAF inhibitors PLX-8394, TAK-580, BGB-283).^217^ However, it remains difficult to overcome the primary or acquired resistance that almost inevitably occurs. In addition, attention should be drawn to the issue that in cells with wild-type BRAF, RAF inhibitors may transactivate ERK signaling resulting in toxicity and drug resistance.^218^ The low occurrence of BRAF V600 mutations in HCC targeted by vemurafenib, dabrafenib, and encorafenib also limits their application value.

### PI3K-AKT pathway

PI3K-AKT has been broadly characterized to be a critical and ubiquitous pathway in regulating the cell cycle. PI3K was discovered to phosphorylate phosphatidylinositol lipids and to act as downstream of RTKs and insulin signaling.^219,220^ PI3K-AKT-mTOR has been found constitutively activated in cancer and acts as oncogenic pathway, regulating cell cycle, survival, metabolism, motility and angiogenesis in cancers.^221,222^ In HCC, activation of PI3K-AKT-mTOR signaling is found in about 50% cases, which is involved in upregulation of EGFR, PI3K, AKT and mTORC1,^223–228^ while the key suppressor of the PI3K/AKT cascade such as TSC1/2 and PTEN were found with loss-of function mutation or reduced expression in HCC.^229^ Activation of PI3K-AKT-mTOR signaling by overexpression of AKT or knockout of Pten/Tsc1/2 were validated to induce HCC hepatocarcinogenesis in mouse models,^230^ powerfully validating the oncogenic roles of this signaling. PIK3C3 is required to the cancer stem cells (CSCs) growth and activity.^231^ Hepatic mTORC2 facilitates hepatosteatosis via de novo fatty acid and lipid synthesis in HCC.^232^ While long-term inhibition of mTORC1 promotes HCC development through promoting IL-6/STAT3 pathway in a murine model of obesity,^233^ which indicates the complex function of mTORC1 in HCC might relates to the etiology. In addition to the initiation of HCC, PI3K-AKT pathways have been broadly investigated to facilitate HCC progression via metabolic reprogramming, such as glucose metabolism, lipid metabolism, amino acid metabolism, pyrimidine metabolism, and oxidative metabolism.^234^ HCC patients with altered PI3K-AKT-mTOR signaling showed the activation of asparagine synthetase (ASNS), glycolysis, and the pentose phosphate pathway.^235^ The significant tumor-promoting roles of PI3K-AKT signaling were substantially demonstrated as above, so the anti-tumor effect by suppression of this pathway has been frequently clarified in HCC, and several agents were under the investigation of clinical trials.^230^

Several selective inhibitors targeting PI3K-AKT-mTOR cascade have progressed in phase II studies for the treatment of liver cancer, of which the outcome is inconsistent and with significant adverse effects. Copanlisib, a specific PI3K inhibitor, was evaluated in cancer patients with activating mutation of PI3K in a phase II clinical study (NCT02465060).^236^ Another phase II study evaluated the efficacy of an allosteric AKT inhibitor, MK-2206, in advanced biliary cancer (NCT01239355).^237^ While the two trials showing discouraging results with limited efficacy and severe adverse effects.^236,237^ Though a phase I/II study failed to exhibit effectiveness of single RAD001 (everolimus) targeting mTOR for treating patients with advanced HCC (NCT00516165), another mTOR inhibitor sirolimus (rapamycin) was encouragingly demonstrated to improve the survival of HCC patients in 2 phase II studies.^238^ An orally administrated mTOR inhibitor, ATG-008 (CC-223) was tested in HBV-positive HCC in a phase II trial (NCT03591965), while terminated based on strategy development. An ATP-competitive mTOR kinase inhibitor AZD8055, has undergone an evaluation of its safety, tolerability, pharmacokinetics, and preliminary efficacy in a phase I/II study, (NCT00999882).^239^ Several clinical trials are ongoing to explore drug combinations of inhibitors targeting PI3K-AKT-mTOR signaling cascade.^230,240^ The combination treatment of rapamycin with bevacizumab demonstrated complete response (CR) in 1 case, PR in 2 cases, and stable states of the disease in 14 cases out of 20 evaluable HCC patients in a phase I study (NCT00467194).^241^ A single-arm phase II trial exploited the inhibition of mTOR with temsirolimus along with sorafenib’s effects on HCC,^242^ showing on improvement in overall survival compared with the treatment of single sorafenib (NCT01687673). Overall, therapies targeting this pathway alone or connectionally showing promising anti-HCC potential in clinical assessment, but it still requires further investigation to improve the clinical response and avoid toxicities and adverse effects.

Except for the limited agents under clinical assessment, the preclinical research of other agents targeting this signal axis is continuously attracting attention. These agents include potent PI3K inhibitors (LY294002, DZW-310,^243^ 740Y-P,^244^ copanlisib^245^) and AKT inhibitors (MK2206, AKT inhibitor VIII).^246,247^ These agents elicited consistent anti-tumor effectiveness such as proliferation inhibition and apoptosis induction in HCC cells.^230,248,249^ Other unselective agents have also found to inhibit HCC.^230,250^ For example, celecoxib, a non-steroidal anti-inflammatory drug, targets the cyclooxygenase 2 (COX-2)/AKT pathway and was sufficient to inhibit the progress of HCC by inhibiting lipogenesis.^250^

Activation of PI3K/AKT/mTOR signaling also associates with sorafenib resistance in HCC,^251^ providing the rationale for the combination of targeting PI3K/AKT/mTOR cascade with sorafenib. For instance, PI3K inhibitor copanlisib exhibits synergistically anti-tumor effectiveness to sorafenib in HCC treatment,^245^ the synergistic effectiveness was also observed in the treatment with lenvatinib.^248^

### JAK-STAT pathway

JAK-STAT pathway is an evolutionarily conserved signaling transduction pathway and functions in cell proliferation and survival, modulation of the immune response, as well as angiogenesis and metabolism.^252–254^ Nearly 60 cytokines including various interleukins, growth factors and colony-stimulating factors (CSFs) act as ligand to activate this pathway.^252^ Upon binding to the receptor, JAKs activate STATs by phosphorylation, which provokes STATs dimerization and subsequent translocation to the nucleus where they stimulate the transcription of specific target genes responsible for immune system development, immune regulation and hematopoiesis.^253,254^ The vital role of JAK-STAT pathway is well-known pivotal to neoplastic disorders including hematopoietic and solid cancers.^252^ Several studies affirm aberrant activation of this pathway and its promotion of malignant phenotypes in HCC via different mechanisms. Actually, STAT3 was indicated to be constitutively active in nearly 60% of the HCC cases.^255^ Activation of STAT3 causes the transcriptional expression of genes associated with the diverse hallmarks of cancer, like Cyclin D in cell cycle, VEGF in angiogenesis, and IL-10 in immunosuppression, indicating the crucial roles of STAT3 in HCC.^256^ In 9% of HBV-related HCC cases, missense mutations in JAK1 were found and led to activation of JAK1 and STAT3, allowing cytokine-independent growth.^257^ In contrast, the negative regulators of the JAK/STAT pathway, CIS, SOCS1 and SOCS3 were found frequently downregulated or lost in HCC, thus resulting in continuous activation of the pathway and HCC progression.^258–262^ The diversity of ligands and receptors, as well as JAKs and STATs can activate the JAK-STAT pathway, which contributes to its complexity and various cellular responses in cancers. Namely, STAT5A/B act as protumor factors like STAT3, while STAT1 and STAT2 present antiproliferative effects in HCC both in vitro and in vivo.^263–265^

As an essential immune-regulated pathway, interferon-alfa (IFN-α) mediated JAK-STAT signaling induces various target genes with antiviral and immunomodulatory functions, based on which IFN-α was used to drive host antiviral responses as the current first-line therapy for chronic hepatitis B and has been confirmed to slow the progression of liver fibrosis and even the emergence of HCC,^266^ indicating the promising characteristics of this pathway in HCC therapy. Actually, kinds of JAK/STAT inhibitors have been examined for their clinical significance in diverse cancers, including HCC, mainly focused on JAK and STAT3 inhibitors.

As the upstream of this signaling axis, JAKs function as a feasible target to curb the downstream effects. WP1066,^267,268^ pacritinib,^269,270^ common JAK inhibitors like cryptotanishinone and ruxolitinib are being studied in human diseases. However, these compounds are still in preclinical stages for HCC treatment.^271–273^ For example, WP1066 has been demonstrated to inhibit MMPs and counteract the activity of UCK2, which suppressed the migration and invasion abilities of HCC cell lines.^274^

STAT3 can be directly suppressed by small molecule compounds like static,^275^ OPB-111077,^276^ OPB-31121,^277^ napabucasin ^278^, and siRNA (AZD9150).^279^ OPB-111077 was well tolerated overall while showed limited efficacy in sorafenib-refractory HCC patients.^276^ OPB-31121 in a phase I research of advanced solid tumors (NCT00657176) and HCC (NCT01406574)^277,280^ showed limited antitumor efficacy with toxic side effects. A phase Ib/II clinical trial is evaluating napabucasin in combination with sorafenib for HCC (NCT02279719). Another Phase III trial is also underway for the combination of napabucasin and paclitaxel in gastric and gastroesophageal junction cancers (NCT02178956).^278^ Unlike small molecule inhibitors, AZD9150 is a siRNA targeting STAT3, showing clinically valuable antitumor activity and can be regarded as a safe treatment for diffuse large B-cell lymphoma.^281^ A phase I study showed that AZD9150 was well tolerated with mild and a few serious adverse events (NCT01839604) in HCC.^279^ Nevertheless, further studies are required to illuminate its clinical efficacy. Some agents showed therapeutic effect by indirectly targeting STAT1. An example is acyclic retinoid acts synergistically with IFNs in suppressing the proliferation of HCC cells in vitro by increased expression and DNA-binding activity of STAT1.^282^

Altogether, these studies reveal the potential of targeting the JAK/STAT pathway in HCC. While clinical research on these inhibitors are still in the initial phases for HCC and adverse effect need attentions. The beneficial effects observed in other tumor types offer indications of potential clinical efficacy for HCC likewise.

**Fig. 2:**
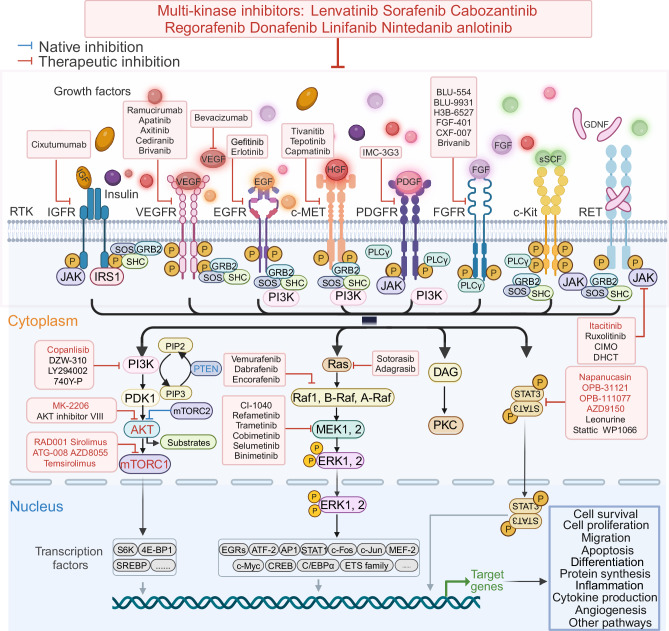
Summary of the RTK signaling pathways and their related inhibitors in HCC. The RTK and downstream signaling pathways are described, along with a list of inhibitors targeting single or multiple targets. Sorafenib and donofenib inhibit VEGFR1–3, PDGFR, RAF, KIT; lenvatinib inhibits VEGFR1-3, PDGFR, FGFR1-4, RET; cabozatinib inhibits VEGFR1–3, MET, RET; rogerafenib inhibits VEGFR1–3, PDGFR, RAF, FGFR1-2; linifanib inhibits KDR, Flt-1, PDGFRβ, and FLT3; nintedanib inhibits VEGFR1/2/3, FGFR1/2/3, and PDGFRα/β; anlotinib inhibits VEGF R2/3, FGF R 1-4, PDGF R α / β, c-Kit, and Ret. Figure was created with biorender.com

Although the frequency of mutations in RTKs is not high, they were detected to be overexpressed in HCC tissues of most patients and are closely associated with malignant biological behaviors such as tumor angiogenesis, proliferation, invasion, metastasis, and drug resistance. Therefore, most RTKs genes are considered as oncogenic driver genes. A series of tyrosine kinase inhibitors and antibody-based agents have been developed and tested as shown in Fig. 2.

### Other pathways in HCC

Beyond the frequently altered and therapeutically targeted RTK pathways in HCC. Pathways in cell differentiation such as Wnt/β-catenin, Hippo, Hedgehog and Notch are especially important for liver tissue homeostasis, and closely correlates to liver disease including HCC. Here, we review the main dysregulated signaling pathways with therapeutic advances in HCC (Table 2).

**Table 2:** Summary for therapies targeting signaling pathways in clinical trials for HCC

Signaling path way	Target	Agent	Clinical stage	Setting	Patients	Outcome	Safety	Trial identifier	Refs
PI3K/AKT/mT OR pathway	Pan-class1PI3K	Copanlisib	Phase II	Arm Z1F	Tumor with PIK3CA mutations	ORR of 16% (P5.0341)	Hyperglycemia, fatigue, diarrhea, hypertension, nausea 53% grade 3 toxicities; 3% grade 4 toxicities	NCT02465060	^236^
	AKT	MK-2206	Phase II	Single agent	Advanced liver cancer (biliary cancer) did not respond to previous therapy	No clinical activity	Grade1/2 toxicities (lymphopenia, rash, fatigue, fever, vomiting, diarrhea)	NCT01239355	^237^
	mTOR	RAD001 (everolimus)	Phase I/II	Single agent	Advanced HCC, iCCA	No significant difference	Anemia, Diarrhea, Lymphopenia, Stomatitis, Vomiting, Nausea, Constipation, Pain, Fatigue, et al.	NCT00516165 NCT01035229 NCT00973713 NCT00390195	^646–648^
	mTOR	Sirolimus (rapamycin)	Phase II	Single agent	Liver Transplantation for HCC(immunosuppressive)	Does not decrease HCC recurrence but prolongs OS after L DLT for HCC	Wound complication and dyslipidaemia, acute cellular rejection	NCT01374750	^238^
	mTOR	AZD8055	Phase I/II	Single agent	Patients With Advanced Stage HCC and With Mild or Moderate Hepatic Impairment	Completed	NA	NCT00999882	^239^
	mTOR	Sirolimus	Phase I	Combined with bevacizumab (target VEGF)	Unresected Liver cancer	No survival benefit over bevacizumab-only treatment	Grade 3 thrombocytopenia and grade 3 mucositis. hyperglycaemia (83%), thrombocytopenia (75%), fatigue (46%), mucositis (46%), anorexia (42%), diarrhea (33%) and proteinuria (12.5%)	NCT00467194	^241^
	mTOR	Temsirolimus	Phase II	Combinated with Sorafenib	Advanced Hepatocellular Carcinoma	Acceptable safety but did not achieve the target threshold for efficacy	Grade 3 (hypophosphatemia, thrombocytopenia, and rash)	NCT01687673	^242^
	mTOR	Temsirolimus	Phase I/II	Single agent	HCC	Disease stabilization (defined as CR + PR + SD > 12 weeks) in tumors having high and low pMTOR H-scores to be 70% and 29% respectively (OR 5.667, 95% CI1.129–28.454, *p* = 0.035)	Grade ≥ 3 that occurred in > 10% of patients included thrombocytopenia (4) and hyponatraemia (4)	NCT00321594 NCT01251458 NCT01567930	^649^
	TORC1/2	Onatasertib (CC-223)	Phase II	Single agent	HBV-positive advanced HCC;Advanced solid tumor including HCC	Preliminary antitumor activity	Diarrhea (60.38%), hyperglycaemia (60.38%), thrombocytopenia (30.19%), hyperbilirubinaemia (11.32%)	NCT03591965 NCT01177397	
TGF-β pathway	TGF-βRI	Galunisertib	Phase II	Combined with sorafenib	Advanced HCC and Child-Pugh A liver function without prior systemic therapy	Median OS: 18.8 months;ORR: 4.5%	The most frequent treatment emergent adverse events of any grade: palmar-plantar erythrodysesthesia (56.8%), diarrhea (43.2%), and pruritis (25.0%)	NCT01246986	^292^
	TGF-βRI	Galunisertib	Phase I	Combined with stereotactic body radiotherapy	Advanced HCC who progressed on, were intolerant of, or refused sorafenib	Median OS: 9.0 months; median PFS: 3.7-months; ORR: 14.2%	The most common treatment-related adverse events were fatigue (53%), abdominal pain (46.6%), nausea (40%), and increased alkaline phosphatase (40%)	NCT02906397	^187^
	TGFβ1/GARP	Livmoniplimab	Phase I/II	Combined with Budigalimab	Locally advanced or metastatic and/ or unresectable HCC	Not reported	Not reported	NCT06109272	Not available
Wnt/β-catenin pathway	PORCN	CGX1321	Phase Ib	With Pembrolizumab or Encorafenib + Cetuximab	Advanced GI Tumors	Unkwown	NA	NCT02675946	
	PORCN	CGX1321	Phase I	Dose Expansion	Advanced GI Tumors	Unkwown	NA	NCT03507998	
	Wnt ligands	OMP-54F28	Phase Ib	Combined with sorafenib	HCC patients	Completed	NA	NCT02069145	
	DKK1	DKN-01, (humanized monoclonal antibody)	Phase I	Combined with gemcitabine and cisplatin	Carcinoma to Primary to the Intra-or Extra-Hepatic Biliary System or Gallbladder	No additional activity beyond historically reported efficacy with gemcitabine/cisplatin alone.	Neutropenia (60%), thrombocytopenia (34%), and anemia (23%)	NCT02375880	^328^
	β-catenin-CBP	E7386	Phase Ib/II	Combined with pembrolizumab or pembrolizumab+lenvatinib	Previously Treated Subjects With Selected Solid Tumors, including HCC	Recruiting	NA	NCT05091346	
	β-catenin-CBP	E7386	Phase Ib	Combined with lenvatinib	Solid tumor including HCC	Recruiting	NA	NCT04008797	
	β-catenin-CBP	E7386	Phase I	Single agent	Solid tumor	Recruiting	NA	NCT03264664	
JAK/STAT pathway	JAK1	Itacitinib	Phase Ib	Single agent	Advanced HCC	Ongoing	NA	NCT04358185	
	STAT3	OPB-111077	Phase I	Single agent	Advanced HCC	Limited preliminary efficacy outcomes	Thrombocytopenia (6%), fatigue (3%), and dizziness (3%)	NCT01942083	^276^
	STAT3	OPB-31121	PhaseI/II	Single agent	Advanced HCC	Insufficient antitumor activity for HCC	Peripheral nervous system-related toxicities	NCT01406574	^650^
	STAT3	Napanucasin(BBI608)	PhaseI/II	Combined with sorafenib	Advanced HCC	No additional effect	No dose-limiting toxicities, diarrhea (83.3%) and palmar-plantar erythrodysesthesia syndrome (66.7%)	NCT02279719 NCT02358395	^651^
	STAT3	AZD9150	Phase I/Ib	Single agent	Advanced/Metastatic HCC	Completed	Abdominal pain; Hepatorenal failure	NCT01839604	
Hedgehog pathway	Gli	Arsenic trioxide (ATO)	Phase II	Single agent	Unresectable metastatic liver cancer	Completed	NA	NCT00128596	
	Gli	Arsenic trioxide (ATO)	Phase II	Single agent	Advanced liver cancer	Terminated	NA	NCT00582400	
	Smo	Vismodegib	Phase I	Single agent	Advanced solid malignancies (include HCC) and hepatic impairment	Completed	Four patients experienced dose-limiting toxicity of hyperbilirubinemia on study: one in the moderate cohort and three in the severe cohort.	NCT01546519	^363^
	Hedgehog	LDE225	Phase Ib	Single agent	Advanced or metastatic HCC and Child-Pugh A/B7 Cirrhosis	Completed	NA	NCT02151864	
Hippo pathway	TEAD	IK-930	Phase I	Single agent	Solid tumors with or without YAP1/ TAZ Fusion Genes or NF2 deficient	Recruiting	NA	NCT05228015	
Notch pathway	γ-Secretase	Ginsenoside (RG3)	Phase I	Combined with TACE	HCC with high expression of Notch1 (*n* = 320)	Completed	NA	NCT02724358	
Telomere regulation	TERT	Telomelysin (OBP-301)	Phase I	Evaluate the Safety and Efficacy, non-comparative	HCC	Improved local control in patients with advanced HCC (SD78%)	Influenza-like illness, pyrexia, fatigue, decreased platelet count, 46 abdominal distension, and anemia	NCT02293850	^446^
Epigenetics	Class I, class IIa and IV HDAC	Resminostat	Phase I/II	Alone or combined with sorafenib	Advanced HCC	Resminostat at the recommended dose plus sorafenib showed no significant efficacy advantage over sorafenib monotherapy	Gastrointestinal disorders, thrombocytopenia and fatigue	NCT02400788 NCT00943449	^466,467^
	Pan-HDAC	Belinostat	Phase I/II	Single agent	Unresected Liver cancer	Tumor stabilization, well tolerated	Abdominal pain, hyperbilirubinemia, increased ALT, anemia, and vomiting, grade 4 anemia	NCT00321594	^652^
	Pan-HDAC	Panobinostat	Phase I	Combined with sorafenib	Advanced HCC,Metastatic and/or unresectable Liver cancer	Terminated	NA	NCT00823290 NCT00873002	
	Pan-HDAC	Vorinostat	Phase I	Combined with sorafenib; combined with chemotherapy	Advanced liver cancer	Ten patients (77%) had stable disease (SD), The median treatment duration was 4.7 months for response-evaluable patients.	Anorexia, dehydration, dysgeusia, fatigue, lymphocytopenia, nausea, and thrombocytopenia	NCT01075113 NCT00537121	^465^
	HDAC	Tefinostat	Phase I/II	Dose escalation	HCC	Completed	NA	NCT02759601	
	DNMT	Decitabine	Phase I/II	Combined with Chemo- or immunotherapy	Patients with Refractory and/or Chemotherapy Resistant Solid Tumorsor B Cell Lymphomas	The lower-dose decitabine treatment resulted in beneficial clinical response and favorable toxicity profiles(The disease control rate (CR + SD rate) was up to 46.67%. The treatment prolonged PFS and OS to 4 and 11 months)	Generally well tolerated. The most commonly reported Aes were hematologic toxicity and gastrointestinal symptoms.	NCT01799083	^463^
	DNMT	Guadecitabine (SGI-110)	Phase II	After failure of prior sorafenib	Advanced HCC	Median OS: 245(148–303); Median PFS:82.5(57–113)	Pain in extremity, Abdominal pain, Febrile neutropenia	NCT01752933	^653^
	DNMT	Guadecitabine(SGI-110)	Phase Ib	Combined with durvalumab	Digestive tumors including HCC	Active, not recruiting	NA	NCT03257761	^654^
P53 signaling	MDM2	ASTX295	Phase I	Dose escalation	Solid tumors with wild-type p53	Active, not recruiting	NA	NCT03975387	
	MDM2	HDM201	Phase II	Different matched targeted therapy	Primary tumor or metastatic lesio solid tumors with specific oncoge advanced/metastatic solid tumors	Recruiting	NA	NCT04116541	
	MDM2	Idasanutlin MT	Phase II	Tumor-agnostic precision immuno-oncology and somatic targeting rational for you (TAPISTRY) platform study	Solid tumors with specific oncogenic genomic alterations or TMB	Recruiting	NA	NCT04589845	
	MDM2	Milademetan	Phase II	Single agent	Advanced/ metastatic solid Tumors	Terminated	NA	NCT05012397	
	TP53 Y220C	PC14586	Phase I/II	Alone and combined with pembrolizumab	Solid tumors with p53 Y220C mutation	Recruiting	NA	NCT04585750	
	Mutant p53	Arsenic trioxide	Phase II	Single agent	Refractory solid tumors with rescuable p53 mutation	Unknown status	NA	NCT04869475	
	WT p53	Ad-p53	Phase II	Combined with immune checkpoint inhibitors	Solid tumor approved for anti-PD-1 or anti-PD-L1 Therapy	Unknown status	NA	NCT03544723	
	Bcl-2	Navitoclax	Phase I	Combined with Sorafenib	Solid tumor with HCC expansion cohort	This combination was tolerable but had limited efficacy in the HCC expansion cohort, with stable disease as best response in 6 (40%) HCC patients.	Thrombo7cytopenia, increased AST, fatigue, increased ALT, diarrhea, increased alkaline phosphatase, and rash.	NCT01364051	^503^
	CDK 4/6	Palbociclib(PD-0332991)	Phase II	Single agent	Advanced HCC	Ongoing	NA	NCT01356628	
	CDK 4/6	Ribociclib(LEE011)	Phase II	Combined with chemoembolization	Locally advanced HCC	Completed	NA	NCT02524119	
	CDK 4/6	Abemaciclib	Phase II	Combined with Nivolumab	HCC	Completed	NA	NCT03781960	

### TGF-β signaling

The transforming growth factor (TGF-β) family is the most diverse and far-reaching family of cytokines in the human body and is expressed in almost all types of tissues and cell types.^283^ This superfamily consists of 33 multifunctional factors, and TGF-β isoforms (I-3) are the typical members, with TGF-β1 being the most abundant and well-studied.^284^ Three isoforms of the TGF-β receptor exist, types I, II, and III, the first two being single transmembrane glycoproteins with serine/threonine kinase activity, and the latter being proteoglycan with no kinase activity.^285^ TGF-β is a major player in tumorigenesis. It has a well-known dual effect in cancer and is no exception for HCC.^286^ For normal tissues, TGF-β signaling inhibits cell proliferation and induces apoptosis or differentiation by blocking cells at the G1 phase or prolonging the G1 phase, thus acting as a tumor suppressor in precancerous cells.^285^ In the early stages of carcinogenesis, TGF-β inhibits tumorigenic inflammation or triggers apoptosis in precancerous progenitor cells carrying RAS mutations. As tumors progress to advanced stages, TGF-β signaling gradually exhibits pro-tumorigenic effects such as induction of immunosuppressive microenvironment, EMT, and promotion of angiogenesis.^283^ In HCC, downregulation of the TGF-β signaling pathway may imply loss of cancer suppressive activity, while upregulation leads to fibrosis and inflammation.^287^ Nearly 40% of HCC patients have somatic mutations in at least one member of the TGF-β pathway.^287^

Increasing preclinical and clinical evidence suggests that TGF-β signaling activation in established anti-cancer therapies such as targeted therapies, chemotherapy, and radiation is a driver of drug resistance,^288,289^ involving multiple mechanisms such as induction of EMT, metabolic reprogramming, activation of alternative pathways, promotion of an immune-suppressive microenvironment, induction of a stem cell-like phenotype, and promotion of drug uptake and efflux.^290,291^ Therefore, the prevailing view is that therapies combining TGF-β signaling inhibitors with other anti-tumor therapies is a promising strategy that not only inhibits advanced tumor progression but also reverses treatment resistance. However, TGF-β receptors and their ligands are widely distributed in vivo, so potential systemic cytotoxicity is a major obstacle. Currently, the only FDA-approved TGF-β inhibitor, liuspatercept, is indicated for anemia in adults with Myelodysplastic Syndromes (MDS). For HCC, a small molecule inhibitor targeting TGFβR1, galunisertib, in combination with sorafenib as first-line therapy for advanced patients has demonstrated a manageable safety profile and improved OS.^292^ The combination of galunisertib with stereotactic body radiotherapy demonstrated tolerable toxicity in a pilot study.^187^ However, galunisertib did not show sensitization to VEGFR inhibitors in HCC patients as shown in preclinical studies.^293^ In addition, a neutralizing antibody, NIS793, was recently tested for safety in advanced malignancies, including HCC, supporting the further exploration.^294^ A phase III trial was recently initiated to test the efficacy and safety of livmoniplimab, a humanized monoclonal antibody against the LRRC32/TGF β 1 complex, in combination with a PD-1 inhibitor in advanced HCC (NCT06109272). Considering the dual role of TGF-β in tumors, targeting the TGF-β pathway may only be effective in specific HCC patients, thus identification of biomarkers is warranted. For example, previous studies have found that upregulation of SMAD7, CLTC, and CXCR4 is associated with the pro-carcinogenic profile of TGF-β, and it may be worthwhile to further test whether clusters with high expression of these genes are more likely to respond to TGF-β inhibitors.^295^

### Wnt/β-catenin signaling

Without the presence of extracellular Wnt ligands, the canonical Wnt signaling is inactive. β-catenin is located in adherent junctions and cytoplasm of the cell, where it becomes phosphorylated by the destruction complex (containing adenomatous polyposis coli protein (APC), Axin, casein kinase I isoform-α (CKIα) and glycogen synthase kinase 3 β (GSK3β) and targeted for ubiquitylation and proteasomal degradation. Wnt signaling is tightly regulated intracellularly by the canonical molecules in this pathway mentioned above. It is also modulated by extracellular antagonists and inhibitors, such as Wnt inhibitory factor 1, Dickkopf-related proteins (DKKs), and other novel potential regulators.^296^ Here, we focused on the role of Wnt/ β-catenin in HCC. Wnt-β-catenin signaling is commonly overactive in HCC. 20–35% of HCCs have genetic mutations and/or aberrant activations of key genes involved in this cascade.^297^ Human HCCs with aberrant Wnt/ β-catenin activation present distinct clinical, pathological, and molecular characteristics. The gain-of-function mutations of CTNNB1 occurred in HCCs with HCV (28%),^298,299^ alcohol (42%) ^70^ and non-cirrhotic liver in the absence of common risk factors of HCC.^300^ While AXIN1 is more frequently mutated in HBV-related HCCs (18%) than in HCV-related or non-viral HCCs (14% and 8%, respectively).^299^ Mutations of Wnt/β-catenin were more often identified to be associated with nonproliferation subgroups of HCC but also found in proliferation group.^300^

Wnt-β-catenin has been connected to HCC stemness, progression, metastasis and drug resistance. As an illustration, elevated activity of β-catenin prominently leads to proliferation, self-renewal and in vivo hepatocarcinogenesis of CSCs in HCC.^301–303^ In transgenic mice models, activation of Wnt/ β-catenin was found to induce HCC formation when cooperated with other-oncogenic signaling such as activated c-Met,^304–306^ K-Ras,^307^ Akt,^308^ LKB1,^309^ and Nrf2^310^. Co-expression of these oncogenes and activation of Wnt/ β-catenin were also found in human HCC tissues.^83,304,310^ The Wnt/β-catenin axis exerts crucial effects in HCC through regulating the downstream target genes. β-catenin induced c-MYC expression^311,312^ is implicated in gankyrin-driven glycolysis, glutaminolysis^313^ and sorafenib responsiveness in human HCC.^314^ Another direct target of this signaling, cyclin D1, was frequently found to be upregulated by activation of Wnt-β-catenin in mouse and human HCC.^315,316^ The interlinked and feedback mechanisms between cyclin D1 and Wnt-β-catenin were involved in hepatocarcinogenesis and HCC metastasis.^300,317^ Other target genes of Wnt/β-catenin such as glutamine synthetase (GS)^318,319^ and KIF2C^320^ were found to link with mTOR cascade and promote HCC growth.^321,322^ Activation of Wnt/β-catenin has also been linked to resistance against Lenvatinib, sorafenib and regorafenib in HCC patients.^302,323,324^ Above all, the activities of the Wnt/ β-catenin cascade during hepatocarcinogenesis have not been clearly characterized and need further investigation.

Porcupine (PORCN) is an O-acyltransferase indispensable for Wnt ligand secretion,^325^ whose inhibitor CGX1321 is tested in patients with advanced solid tumors, like HCC and CCA (NCT02675946, NCT03507998). OMP-54F28, which binds Wnt ligands competing with native Frizzled 8, was assessed in a phase I study in combination with sorafenib in HCC patients (NCT02069145). Dickkopf-1 (DKK1) is known as an extracellular antagonist of Wnt,^326,327^ thus inhibition of DKK1 can suppress β-catenin signaling. DKN-01, a DKK1-neutralizing monoclonal antibody is being evaluated in a phase I trial in combination with gemcitabine and cisplatin in patients with CCA or gallbladder cancer (NCT02375880), but did not show additional activity beyond gemicitabine/cisplatin alone, which may be due to heterogeneity of differential activity for DKN-01 to different DKK1 expression or the need for increased dose/intensity.^328^ Whereas the effect of DKN-01 in HCC keeps unknown.

Niclosamide is a FDA-approved drug and was used to treat taeniasis, showing inhibiting effect on tumors proliferation, stemness and metastasis with limited toxicity in other cancers,^329,330^ providing hopeful optimism for human HCC therapy. Besides, kinds of common drugs in clinic, such as vitamin D and retinoic acid, have been reported to block the interaction of β-catenin with TCF, NSAIDs, such as aspirin and sulindac, have been proved to strengthen the degradation of β-catenin, showing promising potential in preclinical and clinical settings for liver cancer including HCC therapy.^297^

The majority of agents targeting Wnt-β-catenin axis have been evaluating in preclinical studies, providing the rationale for clinical assessment. Kinds of inhibitors showed growth suppressive effect in HCC, including PORCN inhibitors (LGK-974, ETC-159),^331^ fungal derivatives (PKF115-854 and CGP0449090),^332,333^ TNKS inhibitor XAV939.^334^ Interfering RNA– or antisense RNA–based therapy constitutes another method to suppress the Wnt/β-catenin pathway, showing suppressive effect to HCC in vitro^335,336^ and in mouse models.^307,337^

In summary, preclinical studies have assessed the therapeutic prospect of targeting this pathway against cancers, including HCCs. The clinical development of these agents has been rather restricted to date. Therefore, the therapeutic effects for targeting Wnt-β-catenin pathway remain elusive.

### Hedgehog signaling

Hedgehog (Hh) signaling regulates cell proliferation, differentiation, tissue homeostasis, and carcinogenesis.^338,339^ It is composed of the Hedgehog ligand, nuclear transcription factors and two transmembrane protein receptors, Patched-1 (Ptch1) and Smoothened (Smo).^340^ In humans, there are three types of Hedgehog ligands: Sonic Hedgehog (Shh), Indian Hedgehog (Ihh) and Desert Hedgehog (Dhh), which are disparate in the timing of expression, spatial distribution and action characteristics.^341,342^ The role and mechanism for Shh is the most studied.^342^ When Hedgehog ligands are absent, a Glioma-associated oncogene (Gli) is phosphorylated^343–347^ and then undergoes proteolytic cleavage, generating Gli repressor (GliR).^345^ GliR binds to the promoters of Hedgehog target genes and represses their transcription. When Hedgehog ligands bind with the receptors, the canonical Hedgehog signaling pathway is activated.^338^ The binding of Hedgehog ligands to Ptch alleviates the restraint of Smo by the receptor protein, resulting in the movement of Smo to primary cilium (PC).^338,348,349^ The existence of Smo in PC hinders phosphorylation and proteolytic cleavage of Gli, avoiding the generation of GliR. Thus, the promoters of Hedgehog target genes are available to transcription. Hedgehog signaling pathways are widely investigated to crosstalk with the Notch during cell development in various systems and in cancer biology.^350^

The activation and oncogenic effect of Hh signaling were investigated in multiple levels of HCC cell lines, mice models and clinical samples. The increased levels of Shh and its target genes like Ptch, smo and Gli were detected in human HCC tissues and HCC cell lines, suggesting activation of Hedgehog Signaling in human HCC.^351–355^ In detail, Shh is overly expressed in approximately 60% of human HCC, Ptch1 and Gli1 are expressed in over 50% of the tumor.^355–358^ Hepatic activated Hh signaling led to hepatic fibrosis and hepatocarcinogenesis.^357,359^ Hedgehog pathway inhibition reduce HCC growth in cell lines and mouse models.^353,360^ Shh signaling pathway provokes cell migration and invasion of HCC cell through Shh and Gli1.^361,362^ Above all, activation of Hh signaling is oncogenic in HCC. Given its participation in the initiation and development of HCC, Hh targeted therapies could be a hopeful strategy to fight against HCC.

Targeting Gli with arsenic trioxide (ATO) in has been assessed in phase II clinical trials in treating patients with metastatic liver cancer (NCT00128596), and advanced primary carcinoma of the liver (NCT00582400). A Smo inhibitor vismodegib, was assessed in phase I clinical trial includes patients with HCC (NCT01546519). However, the causal relationship between vismodegib exposure and serious adverse events are ascertain due to the high number of patients with advanced HCC with cirrhosis.^363^ Sonidegib is a Hedgehog pathway inhibitor approved for treatment of laBCC (US, EU, Switzerland, and Australia) and metastatic BCC (mBCC; Switzerland and Australia).^364^ While in HCC, it remained in phase I clinical stage (NCT02151864) with unpublished results. Thus, the clinical effectiveness of Hedgehog signaling remains unclear.

Preclinical studies targeting the Hedgehog signaling pathway mainly concentrate on suppressing the activity of Smo. Except vismodegib in clinical assessment, cyclopamine is also a smo inhibitor, which can increase apoptosis and inhibted cells growth and proliferation in diverse HCC cell lines like Hep3B and Huh7.^352,355,365–367^ The anti-tumor effects and impact on drug resistance of various agents targeting Gli have also been assessed. GANT61 is a selective small molecular inhibitor of Gli1 and Gli2-mediated transactivation, displaying anti-tumor effect on cell viability, proliferation and migration in HCC cell lines in vitro and growth in xenograft model, as well as the drug resistance of CD44+ HCC patient derived organoids.^368–372^ Another study showed that Bufalin (Bu), one of the topoisomerase II inhibitors, affecting the expression of Gli1 and Gli3 and exhibits consistent inhibitory effect in Huh7, Hep3B and HepG2 cells.^373^ Inhibition of Gli by RNAi can also effectively reverse sorafenib, 5-FU, doxorubicin, and cisplatin resistance in HCC cell lines.^369^ A previous study generated a polymeric nanoparticle-encapsulated Gli inhibitor HPI-1 (NanoHHI) and demonstrated its tumor growth inhibition and antimetastatic effects in an orthotopic model of human HCC.^374^ Some drugs originally intended for other pathways and diseases have also been found to inhibit the Hh pathway. They can act as unselective inhibitors of the hedgehog pathway and exerts anti-tumor effects in HCC, including ITCZ,^375^ Taccalonolide A,^376^ human sulfatase 2 inhibitor 2,4-disulfonylphenyl-tert-butylnitrone (OKN-007).^377^

### HIPPO/YAP signaling

Hippo signaling was firstly recognized as a crucial determinant for organ size,^378^ emerging as a vital player in liver biology, such as liver development, cell fate determination, homeostasis and regeneration from injury.^379^ The cascade is initiated by neurofibromatosis 2 (NF2) and kidney and brain expressed protein (KIBRA) when transmembrane receptors transduce signals to them. The canonical Hippo signaling pathway acts to suppress the activity of YAP/TAZ. When the Hippo pathway is inactivate, the abolition of YAP phosphorylation by MST1/2 enables YAP to translocate into the nucleus, subsequently interacting with the transcription factor TEAD to mediate the target genes expression such as RUNXs, SMAD, PPARG.^379^ Hippo/YAP signaling exerts a deep impact on the physiopathology of liver diseases including hepatic malignancies. In HCC, Hippo signaling is involved in various oncogenic effects such as cell proliferation and apoptosis, cell cycle and differentiation. YAP activation is an early occurence in HCC development. Genomic amplification of the genomic locus which contains the YAP gene (11q22) is found in 5–10% of HCC,^380^ and increased YAP activity is found in 65–85% of HCC.^381,382^ Activation of YAP is associated with more aggressive subtypes of HCC and is an independent prognostic marker in HCC.^383–385^ Activation of Hippo acts as tumor suppressor, while YAP/TAZ activity has shown to promote carcinogenesis in HCC. For instance, Nf2-deletion, hepatic inactivation of HPO1/2 lead to generation of HCCs, iCCAs (intrahepatic cholangiocarcinomas), and mixed hepatocellular cholangiocarcinomas.^386–389^ Both YAP and TAZ are required for c-Met/sgAxin1-dependent hepatocarcinogenesis using conditional knockout (KO) mice.^390^ Ablation of TAZ completely prevented c-MYC-induced hepatocarcinogenesis in knockout mice.^391^ Multiple regulators participate in the oncogenic activity of YAP in HCC, such as NUAK2,^392^ Notch signaling,^393^ Ccl2 related immune regulation.^394,395^ Other signalings can interact with Hippo pathway to promote HCC, such as Notch pathway, EGFR signals, AKT pathway and DDR1 signaling^396–399^

Considering the oncogenic roles of YAP in liver cancer and other disease, it is attractive to develop inhibitors targeting YAP. However, the currently developed selective YAP activity inhibitors were clinically evaluated in other cancers such as non-small cell lung cancer and gastric cancer rather than HCC.^391^ Several agents target other pathways were found indirectly regulate Hippo/YAP signaling and against HCC, such as Statins (fluvastatin and simvastatin),^400^ Salvianolic acid B (Sal B)^401^ and Tankyrase inhibitor G007-LK.^390^ However, above unselective agents may exist potential off-target toxicity. More selective strategies have been developed to decrease potential off-target effects. For example, NUAK2 has been identified as a critical downstream target of YAP during liver tumorigenesis, and pharmacological inhibition of NUAK2 suppressed YAP-driven tumor growth in vivo.^402^ NIBR-LTSi, a selective small-molecule LATS kinase inhibitor was characterized to activate YAP signaling and blocks differentiation in vitro and in vivo, also accelerate liver regeneration following extended hepatectomy in mice,^403^ suggesting a clinical potential after resection therapy for HCC. However, the adverse effect due to the off-target effect and the risk for promoting proliferation of HCC cells need further investigations. A phase I clinical trial of assessment for IK-930, an oral TEAD inhibitor targeting the Hippo pathway in advanced solid tumors was ongoing (NCT05228015). Above all, the clinical assessment of agents targeting Hippo signaling in HCC is lacked and the off-target effects requires great attention.

### Notch signaling

**Fig. 3:**
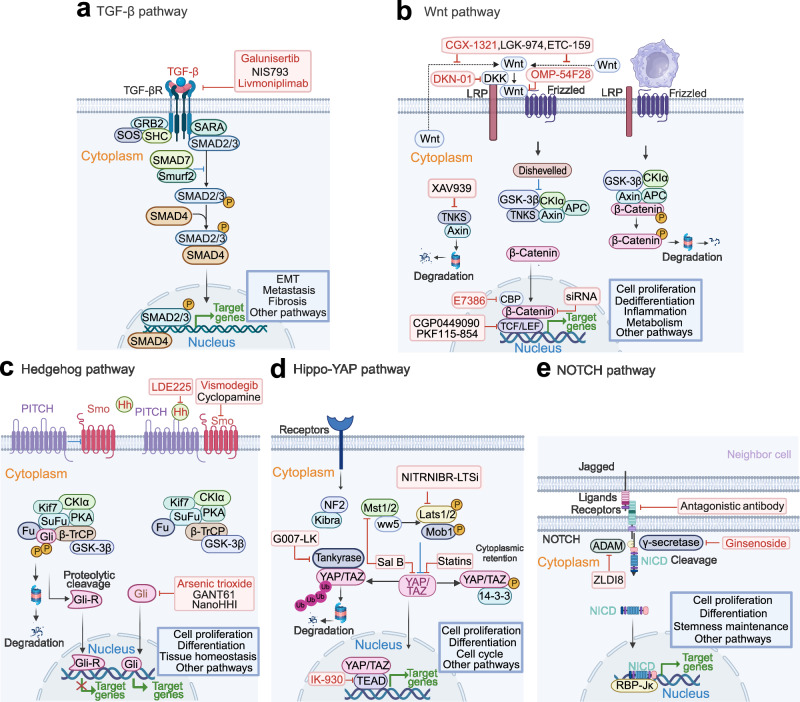
Targeting the other critical signaling pathways in HCC cells. Canonical pathways and pharmacological inhibitors under investigation in HCC for TGF-β pathway (**a**), Wnt pathway (**b**), Hedgehog pathway (**c**), Hippo pathway (**d**), Notch pathway (**e**). The red fonts indicate agents under clinical assessment in HCC patients, the black fonts indicate agents under preclinical investigations. Figure was created with biorender.com

The mechanisms of the canonical and non-canonical Notch signaling pathways have been reviewed previously.^404,405^ Briefly, Notch signaling is initiated through the binding between a transmembrane receptor and a membrane-spanning ligand on adjacent cells. The major components and distinct steps in the Notch pathway have been widely investigated and are summarized briefly (Fig. 3e). Non-canonical Notch signaling can be started by a non-canonical ligand, or in the absence of a ligand, or may not need cleavage of the Notch receptor, or interactions with other cytoplasmic or cytosolic effectors.^406,407^ In addition, Notch signaling crosstalks with others like NF-κB, mTORC, TGF-β, AKT, Wnt, or Hippo to modulate target gene transcriptions.^408–412^ Notch pathway can function as both carcinogens and tumor suppressors depending on the context of cancers and cell populations. Abnormal activation of wild-type Notch signaling and its role in carcinogenesis and progression can be observed in HCC^413^ and other aggressive tumors.^414–416^ The oncogenic function of activated Notch signaling were more frequently found in HCC.^417–419^ Notch signaling is overactivated in human HCC and mouse HCC models, downregulation of Notch1/Jagged1 signaling attenuated HCC progression in mice.^420,421^ Notch signaling also promotes stemness, poor differentiation, epithelial-mesenchymal transition and tumor metastasis in HCC.^422,423^ Notably, inhibiting Notch activity using DAPT causes the differentiation of HCC CSCs into functional hepatocytes via mesenchymal–epithelial transition, indicating that targeting Notch in CSCs may be applied as differentiation therapy for HCC.^424^ Similarly, blocking canonical Notch signaling or treatment with the anti-Notch2 antibody in AKT/Ras mice led to the development of well-differentiated HCC and loss of intrahepatic cholangiocarcinomas-like lesions.^425,426^

Nevertheless, pan-Notch blockade in the triple-knockout mice by using of DAPT resulted in accelerated HCC development and higher expressions of Notch-related genes, thereby supporting a tumor-suppressive role for Notch signaling in HCC.^424^ Recent work indicates that a portion of the tumor-suppressive role of Notch in HCC might not be cell autonomous, and could be associated with crosstalk with Wnt in liver specific tumor-associated macrophages (TAMs).^427^

In general, considering the crucial role of Notch in HCC and blockade of the Notch signaling exhibits alleviated effect in HCC progression, it keeps potential to explore clinical therapeutic effectiveness of targeting Notch signaling in HCC. Diverse classes of Notch-targeting therapeutics have been developed during the past decade, and several agents have been evaluated in clinical trials. Here we sum up the most recent efforts in drug discovery and development targeting Notch signaling in HCC. Present targeted strategies in the clinic mainly converge on modulating γ-secretase and blocking ligand-receptor interaction via related monoclonal antibodies.^428^ γ-secretase inhibitors (GSIs) were the first and most extensively studied small molecule Notch inhibitors, which were initially developed for Alzheimer disease (AD),^429^ then were used as anti-tumor agents due to the target of Notch signaling.^430^ Nirogacestat was approved in the USA for use in adult patients with progressing desmoid tumors who require systemic treatment. However, no clinical advance in HCC.^431^

γ-Secretase modulators (GSMs) are small molecules that keep part of Notch signaling function by modifying the catalytic activity of γ-secretase.^432^ Ginsenoside (Rg3), a natural product from Panax ginseng with GSM properties,^433^ exerts anti-tumor effect by inhibiting NOTCH-Hes1 signaling.^434,435^ It is the only agent targeting Notch signaling has been assessed in a phase I trial (NCT02724358). The result showed that the combination of Rg3 and TACE prolonged OS than TACE monotherapy in HCC patients with high NOTCH1 expression.^436^ Later, Rg3 presented a synergistic anticancer effect for sorafenib in HCC cell lines and mouse model,^437^ which needs further preclinical and clinical exploration. ADAM proteases act as ‘sheddases’ for a great many of membrane proteins including Notch receptors and Jagged 1. Blocking Notch pathway by ADAM17 inhibitor ZLDI-8 may sensitize HCC cells to sorafenib in vivo and in vitro by affect crosstalk between the Notch1 and Integrinβ/ILK signaling pathways in HCC in vivo and in vitro.^438^

Despite the limited therapeutic advance targeting Notch signaling, it keeps attractive to evaluate the clinical therapeutic effectiveness of this signaling combined with other approved treatments in HCC. Taking into account the multiple roles in the development and homeostasis of the immune system, the pharmacological manipulation of Notch has promising applications in cancer immunotherapy.

### Telomere regulation pathway

Telomeres exist in all mammalian cells and their shortening with cell proliferation promotes cell arrest, senescence and apoptosis. Telomeres terminate with a 50–200 nucleotide of TTAGGG repeats single-stranded 3′ overhang that can invade preceding telomeric dsDNA to form a stable telomere loop (T-loop) structure with shelterin at each end of chromosome, which prevent against the loss of telomeres during cell division.^439^ Telomeres are produced by telomerase, which consists of telomerase reverse transcriptase (TERT), the telomerase RNA component (TERC), and dyskerin.^440^ TERT uses an TERC as RNA template to synthesize single-stranded TTAGGG repeats and has a crucial role in telomere maintenance. TERT levels typically act as the limiting factor for telomerase activity in somatic human cells.

Telomere attrition acts as a barrier to replicative immortality, dysfunction of which enables cancer cells to overcome the replicative death, thereby providing a rationale for its therapeutic application in cancer.^439,441^ As the most prevalent somatic mutations, TERTp was observed mutated in up to 60% of human HCC and has been proved to closely relate to sequential hepatocarcinogenesis. The reactivation of TERT mainly through various mechanisms such as mutations in the chromosomal rearrangements (5–10%), promoter (30–60%) and gene amplification, it results in a re-expression and enhanced activity of TERT, which boosts cell survival and proliferation, and against senescence.^440,442^

Targeting telomerase have been developed and assessed as new therapies in HCC. When the expression of TERT was silenced with antisense oligonucleotides in human liver cancer cell lines and in xenograft mouse models, it led to proliferation arrest and death of tumor cells. This provided preliminary evidence for the therapeutic potential of TERT inhibition in HCC.^443,444^ BIBR1532, a small molecule competitively blocking the active site of telomerase, has been linked to telomerase inhibition and HCC cell death in preclinical studies.^445^ However, there is no data in late phase clinical trials.

There are no agents directly targeting TERT in HCC clinical trial. The only clinical application is Telomelysin (OBP-301), a TERT-driven oncolytic adenovirus that specifically introduces the TERT promoter into tumor cells, which is being investigated in a phase I trial in HCC.^446^ In this study, the therapeutic efficacy of OBP-301 was less than that of other second-line systemic targeted therapies. However, OBP-301 injection increased infiltration of CD8+ T cells and <1% PD-L1 expression in tumor, suggesting its potential in combination with other immuno-therapeutics.

**Fig. 4:**
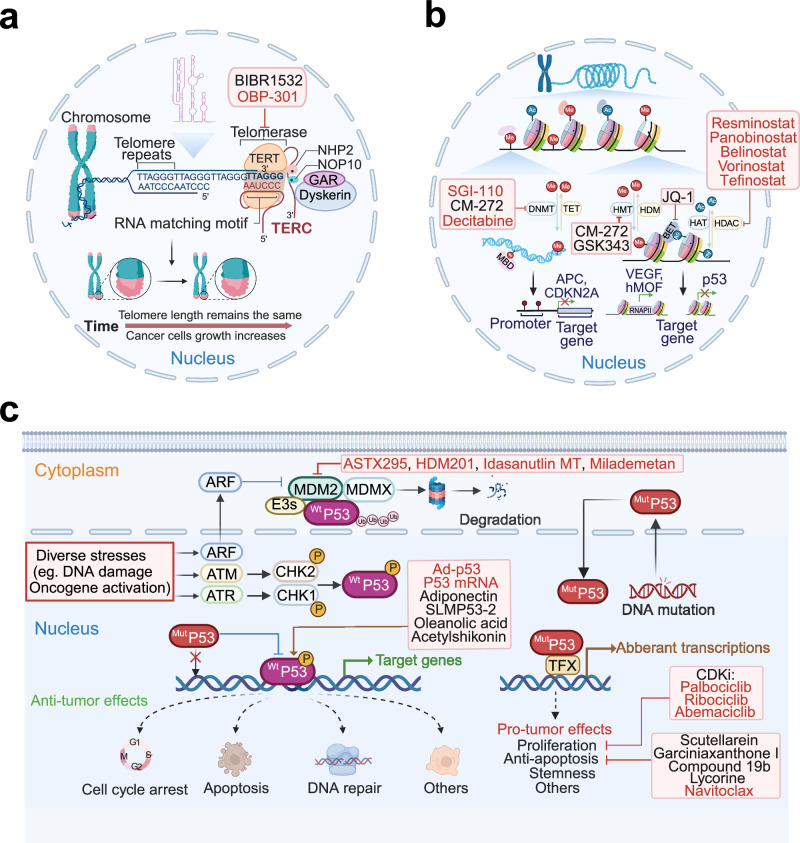
Nuclear signaling pathways. Canonical pathways and pharmacological inhibitors under investigation in HCC for pathways intersecting with nuclear signaling. **a** Telomere regulation pathway. **b** Epigenetic modification. **c** P53 regulation and signaling. The red fonts indicate agents under clinical assessment in HCC patients, the black fonts indicate agents under preclinical investigations. Figure was created with biorender.com

Conclusively, inhibitors targeting the TERT pathway have been studied in cellular and murine models of HCC (Fig. 4a); however, they have not yet undergone clinical trials for therapeutic evaluation, leading to elusive therapeutic effects. Currently, the only related product in clinical trials is the oncolytic adenovirus OBP-301, which exploits TERT promoter activity to achieve anti-tumor effects through virus replication. Nevertheless, its therapeutic effects appear inferior to those of second-line targeted agents.

### Epigenetic pathways

Epigenetic modifications are inheritable variations in gene activity that do not change the DNA sequence.^447^ The two primary types of epigenetic modifications are DNA methylation and histone post-translational modifications (PTMs). DNA methylation occurs mainly at CpG sites, primarily found in CpG islands within regulatory regions of genes. DNA methyltransferases (DNMTs) introduce methyl groups, while ten-eleven translocation enzymes (TET1/2/3) actively remove them. Global hypomethylation of DNA is common in cancer, promoting chromosomal instability and reactivation of endogenous retroviral sequences.^448^ The analysis based on DNA methylation profiles of 304 HCC patients discovered a CpG methylation signature that is correlated with patient survival, among which, IGF, PI3K, TGF-β and WNT pathways were mostly dysregulated in HCC by DNA methylation.^449^ DNA is wrapped around histone proteins to form nucleosomes, where histone proteins undergo various PTMs such as acetylation, methylation, phosphorylation, and more. These modifications, along with DNA methylation, regulate chromatin conformation and accessibility to regulatory elements. Histone methyltransferases and demethylases, such as EZH2, EHMT2, SETDB1 and SETD2 were found to be correlated with the clinical characteristics of human HCC tissues.^450^ EZH2-mediated H3K27me3 signifies a major oncogenic chromatin modification and is involved in sorafenib resistance and HCC growth.^451–453^ Other specific histone modifications are also dysregulated in HCC. For instance, the total levels of H3K9me2 and H3K9me3 are commonly found higher in HCC tissues and positively correlates with tumor differentiation and poor prognosis.^454^ Further, specific DNA methylation and histone methylation can act as diagnostic biomarkers in HCC screening.^455^ Histone acetylation, regulated by histone acetyltransferases (HATs) and histone deacetylases (HDACs), is associated with gene activation, while histone deacetylation is correlated to gene repression and silencing.^456^ Histones acetylation and deacetylation were dysregulated and play important roles in HCC progression via modification enzymes. An instance is MOF, a HAT, can promote HCC growth and vascular invasion via acetylating histone H4K16.^457,458^ The expression of the acetylated H3 and H4 reader BRD4 is also elevated in HCC.^459,460^

Unlike genetic changes, the epigenetic modifications are reversible and have a more extensive impact on gene expression than genetic changes, providing potential new targets with more efficacy and safety.^461,462^

Epigenetic drugs in clinical trials for HCC include DNMTi and HDACi, most of which are in combined with other therapies. DNMT inhibitor Guadecitabine (SGI-110) (NCT01752933) and decitabine (NCT01799083) were assessed in phase I/II trial for pretreated patients with advanced HCC, in which decitabine showing beneficial clinical response and favorable toxicity profiles.^463^ A phase Ib evaluation for combination of guadecitabine with PD-L1 antibody durvalumab (NCT03257761) is underway. Several HDACs inhibitors are approved by FDA for the treatment of hematological malignancies, while remained in early phase clinical trials for HCC. For instance, Belinostat (PDX-101) exhibited tumor stabilization and generally well-tolerated in phase I/II study (NCT00321594).^463^ Belinostat also achieved complete tumor rejection in a murine HCC model when combined with CTLA-4 and PD-1 blockades.^464^ Another HDACi tefinostat was assessed in phase I/II clinical test (NCT02759601). Other phase I/II trials also tested the effect of HDACi when combined with sorafenib, including panobinostat, resminostat and vorinostat (NCT00823290, NCT00873002, NCT00943449, NCT02400788, NCT01075113). Vorinostat in combination with chemotherapy (FOLFIRI) was evaluated in patients with digestive cancers including HCC (NCT00537121). However, the results of the completed trials among above did not show significant and clear improvement over sorafenib while led to some toxicity in patients.^465–467^ Other agents and combined treatments showed therapeutic potential in HCC cell lines or murine models, such as pharmacological inhibition of histone methyltransferase EZH2 by GSK343,^468^ CM272, a dual inhibitor of DNMT1 and G9a,^469–471^ combined inhibition of DNMT and EZH2 by 5-Aza-CdR (DAC) and GSK126.^472^ Notably, a selective HDAC8 inhibitor PCI-34051 was found to stimulate antitumor immunity, and mice treated with combination of PCI-34051 and anti-PD-L1 antibody were safeguarded against subsequent tumor rechallenge and stayed tumor-free for over 15 months.^473^ Overall, above studies underline the potential of combining epigenetic inhibitors and ICIs in HCC treatment. Similar synergistic effect for ICIs were found in other epigenetic modulation agents, including BRD4 inhibitor (JQ-1,^474–476^ i-BET762^477^) and other BET domain inhibitors,^478–480^ as well as lysine demethylase 1A (KDM1A)^481^ and NAD-dependent deacetylase sirtuin 2,^482^ while the mechanisms need further study to provide more clues for therapeutic applications (Fig. 4b).

### Cell death pathway

The p53 protein encoded by *TP53* gene is widely recognized as a tumor suppressor protein that acts as a stress responder and transcription factor.^483,484^ Under physiological and non-stressed conditions, the expression of p53 is kept at a low level by negative regulators, especially MDM2 and E3 ubiquitin ligases.^485^ When stimulated by stress signals, mostly like DNA damage, p53 can transcriptionally regulate the expressions of genes to exert multicomplex functions. The classical and earliest recognized functions include cell-cycle arrest, apoptosis, and senescence, and other functions such as metabolism and ferroptosis.^485^ Mutations of *TP53* typically lead to suppressed activity and occur in the majority of human cancers,^486^ which is observed in 31–36.1% patients with HCC.^20,487^ *TP53* mutations are related with decreased protein levels^488^ and reduced OS in HCC patients.^20,489^ Inactivation of p53 and overactivation of MDM2 are discovered to contribute to the carcinogenesis of HCC in the context of viral infection and metabolic disease, and disrupting MDM2-p53 interaction can liberate p53.^490^

Targeting p53 is highly appealing for the development of anti-cancer drugs. The main strategies encompass small molecular inhibitors restoring its pro-apoptotic activity by inducing the refolding of the mutant p53 or blocking the abnormal degradation of p53 through interruption of MDM2-p53 interactions, as well as reestablishing the expression level and function of p53 with gene therapy.^486,491^ To date, several small molecules have been evaluated in clinical trials for the treatment of solid tumors rather than specifically HCC, including inhibitors blocking the activity of MDM2, such as HDM201(NCT04116541), ASTX295 (NCT03975387), milademetan (NCT05012397), and idasanutlin MT (NCT04589845); small molecules targeting mutant p53, PC14586 (NCT04585750) and arsenic trioxide (NCT04869475); and recombinant human adenovirus gene therapy, Ad-p53 (NCT03544723), which was approved by the China Food and Drug Administration (CFDA) in 2003 as the first gene therapy for treatment of head and neck cancer.^492^ For liver cancer, the combination of Ad-p53 with TACE is reviewed to enhance OS and quality of life compared to TACE monotherapy.^493^ Similar improvements in the clinical outcomes were observed in the combined therapy of Ad-p53 with fractionated stereotactic radiotherapy.^494^

Recently, p53 mRNA delivery by nanoparticles shows potential anticancer effect alone or in combination with immune checkpoint blockade in HCC mouse models.^495,496^ Targeting p53 is typically regarded as suppressing tumors through apoptosis and cell cycle arrest. An example is adiponectin, a wildtype p53 activator that can target p53/TRAIL/caspase-8 axis to attenuate HCC progression.^497^ A new molecule, SLMP53-2, can provoke cell-cycle arrest and apoptosis by activating p53 DNA-binding ability in vitro.^498^ Some new extracts from plants can suppress HCC by promoting apoptosis through activating p53/Bax pathway, such as oleanolic acid and acetylshikonin.^499,500^ Although increasing studies have investigated the therapeutic potential of restoring the pro-apoptotic effect of p53,^486,491,501^ no p53-specific drug has yet been approved by FDA or EMA.^486,502^ A few anticancer agents have been investigated in HCC for their direct targeting of apoptotic pathways through the inhibition of anti-apoptotic BCL-2 family members. For instance, navitoclax plus sorafenib demonstrated limited efficacy a phase I clinical study in patients with solid tumor with HCC expansion cohort (NCT01364051).^503^ Other Bcl-2 inhibitors, ABT-199 and obatoclax were evaluated in preclinical studies in combination with Mcl-inhibitor MIK665 and immune checkpoint inhibitors.^504,505^ Targeting apoptosis is not yet under clinical evaluation. Some agents were found to induce apoptosis in HCC cells by targeting other members of the apoptosis pathway, such as scutellarein targeting Fas/FasL,^506^ Garciniaxanthone I,^507^ Compound 19b and lycorine targeting Cyt-C.^508,509^ P53 also transcriptionally regulates the expressions of cyclins and cyclin-dependent kinases (CDKs), which lead to cell cycle arrest. The dysregulation of CDKs and cyclins is a hallmark of cancer, especially CDK4 and CDK6.

Three CDK inhibitors (palbociclib,^501^ ribociclib^510^ and abemaciclib^511^ are approved by FDA and EMA for the treatment of breast cancer, and exhibited antiproliferation activity in HCC cells and animal models,^512–514^ but have only been tested in clinical trials alone or in combination with Regorafenib and Lenvatinib (NCT01356628, NCT02524119 and NCT03781960) with unavailable results^284^ (Fig. 4c).

There are multiple dysregulated signaling pathways are involved in the development and progress in HCC, except for the well-known RTK signaling pathway (Figs. 3 and 4). These common signaling pathways applied for clinical trial keeps limited (Table 2 and Table 3). Among which, a frequently explored pathway is PI3K-AKT cascade, several inhibitors (Copanlisib, MK-2206, RAD001, sirolimus, Onatasertib, Temsirolimus) have been assessed in Phase II study, though showing limited efficacy. The majority of agents targeting these signaling axis remain in preclinical research and some in phase I study for dosage and safety evaluation. Thus, the main challenges for therapeutically targeting signaling pathway are preliminary effectiveness and toxicity. The lack of effectiveness might be related to the intrinsic and acquired drug resistance, as well as the heterogeneity of HCC.^284^

**Table 3:** Summary for signaling pathways in preclinical stage for HCC therapy

Signaling pathway	Target	Agent	Models	Level	Effect	Refs
PI3K/AKT/mTOR pathway	PI3K	DZW-310	Hep3B, MHCC97H, Hep3B xenograft model	In vitro and in vivo	Attenuate angiogenesis and tumor growth	^243^
	PI3K	Copanlisib	Huh7, HepG2	In vitro	Induce apoptosis, inhibit cell growth, cell cycle arrest	^245,248^
	PI3K	LY294002	MHCC97H, Huh7, Mahlavu	In vitro	Induce apoptosis, suppress cell growth	^244,246,655^
	PI3K	Wortmannin	Huh7, Mahlavu, HepG2	In vitro	Induce apoptosis, suppress cell growth	^246^
	PI3K	Fenofbrate	Hep3B, Huh7, Hep3B xenograft model	In vitro and in vivo	Inhibit cell proliferation and migration, induce apoptosis, inhibit tumor growth	^656^
	AKT	AKT inhibitor VIII	Huh7, Mahlavu	In Vitro	Suppress cell growth, induce apoptosis	^246^
	AKT	Orlistat(FASN) or specific penetrating peptides	Akt knock-in mouse	In vivo	Suppress tumor growth	^657^
	mTOR	RAD001 (everolimus)	Patient-derived HCCs	In vivo	Growth inhibition	^658^
	mTOR	Metformin+sorafenib	HepG2, Bel-7402 xenograft model	In vitro and in vivo	Growth inhibition	^659^
	mTOR	miRNA-199a-3p	Huh7 xenograft model	In vivo	Growth inhibition and survival prolong	^660^
	mTOR	miRNA-99a	SMMC-LTNM	In vivo	Decreased tumor size	^661^
	PI3K/mTOR	BEZ235	Hep3B, Huh7, PLC/RLF5	In vitro	Growth-inhibitory, induce apoptosis	^247^
	AKT/mTOR	Lenvatinib	Hep3B, HepG2	In vitro	Inhibit cell proliferation and migration	^662^
	AKT/mTOR	ZJQ-24	HepG2 xenograft model	In vivo	Inhibits angiogenesis, induce apoptosis and growth inhibition	^663^
	PI3K/AKT	Artemisia rupestris L.(ARL)	HepG2	In vitro	Restrict HepG2 cell proliferation, apoptosis, migration and invasion	^664^
	PI3K/AKT	YangzhengXiaoji capsue (YXC)	MHCC97H, HCCLM3, HepG2, Huh7, liver xenograft tumor model of MHCC 97H and HCCLM3	In vitro and in vivo	Inhibit HCC tumor growth	^665^
	mTOR+VEGF	Rapamycin+bevacizumab	HepG2 xenograft model	In vivo	Growth inhibition and survival prolong	^666^
	PI3K/AKT/mTOR	Suaeda vermiculata Forssk (SVE)	DEN-treated rats	In vivo	Suppress the development of HCC	^667^
	PI3K/AKT/mTOR	Isoliquiritigenin (ISL)	SMMC-7721, MHCC97H, SMMC-7721 xenograft model	In vitro and in vivo	Induce apoptosis and autophagy and tumor growth	^655^
Wnt/β-catenin pathway	PORCN	LGK-974(WNT-974), ETC-159	HepG2 cell lines	In vitro	Enhances radiosensitivity	^668^
	TNKS	XAV939 or WXL-8	HepG2 and HepG2 xenograft model	In vitro and in vivo	Growth inhibition	^334^
	β-catenin/TCF	Fungal derivatives (PKF 115-854, CGP0449090)	HCC cell lines(HepG2, Hep40, Huh7), HepG2 xenograft model Hep3B, MHCC97, and SK-Hep-1	In vitro and in vivo	Growth inhibition	^332,333^
	ß-catenin	RNAi (siRNA)	HuH-6 and HepG2 cell lines	In vitro	Proliferation inhibition	^336^
	ß-catenin	RNAi (siRNA)	HepG2 cell lines	In vitro	Apoptosis and angiogenesis gene expression	^335^
	ß-catenin	LNA-si-β-catenin	GOF Ctnnb1-mutant mouse HCC model induced by diethylnitrosa mine (DEN) and phenobarbita (PB)	In vivo	Decreased cell proliferation and increased cell death	^337^
	ß-catenin	siCTNNB1-LNP	Met/mutant-β-catenin HTV mice model	In vivo	Reduced tumor cell proliferation and viability	^307^
	BCL9 and β-catenin, PD-1/PD-L1	sBBI&PDP nanoparticles	Orthotopic homograft mice model of HCC and a PDX HCC model	In vivo	Antitumor efficacy with a favorable biosafety profile	^669^
JAK-STAT pathway	JAK2	Dehydrocrenatidine (DHCT)	HepG2 xenograft	In vivo	Induced invasion and CSC phenotypes	^670^
	JAK1/2	Ruxolitinib(INC424)	Huh7, SNU182, SNU423	In vitro	Inhibit cell proliferation, promote apoptosis	^272^
	JAK1,2,3	CIMO	Huh7 orthotopic xenograft	In vivo	Reduced tumor growth	^671^
	STAT3	Leonurine	HepG2, Hep3B, and HCCLM3 cells	In vitro	Induced apoptosis and also significantly potentiated the cytotoxic effect of paclitaxe l, doxorubicin, and sorafenib.	^672^
	STAT3	WP1066	Bel7402 cell lines	In vitro	Inhibit cell migration and invasion through EMT	^274,673^
	STAT3	Stattic	HepG2, Bel-7402, SMMC-7721 cell lines	In vitro	Enhance radiosensitivity, reduce radio induced migration and invasion	^674^
	STAT3	Cryptotanshinone (CTS)	MHCC97H,L02 and L02 xenograft model	In vitro and in vivo	Inhibit cell migration, invasion, carcinogenesis;	^675^
			Hepa1-6 model		Inhibit tumor growth and activation of macrophages and polarized mouse bone marrow-derived macrophages (BMM) toward anM1 phenotype; synergy with anti-PDL1	^676^
	STAT3	Napabucasin	Hepa1-6 xenograft	In vivo	Reduced tumor growth	^677^
	JAK/STAT	AZ960, TG101209	Akt/β-catenin hepatic tumor mouse model and tumorspheres	In vivo and in vitro	Abrogate cell proliferation, n regulating the self-renewing	^678^
	JAK1, STAT3	CoL-EtOH	HepG2 cell lines	In vitro	Impeded the viability	^679^
	JAK1, STAT3	Nitidine chloride	HepG2 xenograft	In vivo	Reduced tumor volume	^680^
	JAK2/STAT3	Etomidate	HepG2 xenograft	In vivo	Inhibition of tumor growth Increase in animal survival	^681^
	EphB4 receptor(JAK2, STAT3)	Cantharidin	SMMC-7721	In vivo	Reduced tumor volume	^682^
	IL-6 (STAT3)	miR-515-5p	HepG2 xenograft	In vivo	Inhibit migration and invasion	^683^
	miR-221-3p(STAT3)	C1QTNF1-AS1	HepG2 and Huh7 xenograft	In vivo	Reduced tumor volume	^261^
Hedgehog pathway	Smo	Vismodegib (GDC-0449)	HBxTg mice, Orthotropic Transplantation mice model of hepatoma Mistheton Lectin-1 cell	In vivo	Suppress hepatic tumor development	^684,685^
	Smo	Cyclopamine	HCC cell lines (Hep3B, Huh7, PLC/PRF5), Orthotropic Transplantation mice model of hepatoma Mistheton Lectin-1 cells	In vitro and in vivo	Decreased tumor size	^367^
	Gli	GANT61	Human HCC cell lines and xenograft model of Huh 7, cd44+Patient-derived organoids	In vitro and in vivo	Inhibit proliferation, migration, in vivo growth, drug resistance(sorafenib, 5-FU, doxorubicin, and cisplatin resistance)	^368–372^
	Gli	RNAi	Huh7 cell lines	In vitro	Reverse sorafenib, 5-FU, doxorubicin, and cisplatin resistance	^369^
	Gli	NanoHHI	HCC cell lines(Huh7,MHCC97L), subcutaneous and orthotopic xenograft model of Huh7	In vivo	Growth inhibition and antimetastatic effects in vivo	^374^
	SULF2	OKN-007	HCC cell lines (Huh7, Hep3B) and Huh7 xenografts	In vivo and in vitro	Promoted tumor cell apoptosis and inhibited cell proliferation, viability, and migration, inhibit in vivo tumor growth	^377^
	Shh, Smo and Gli1	Taccalonolide A	HCC cell lines	In vitro	Reduce viability	^376^
	Unselective (Shh, Gli, Smo, etc)	Itraconazole	DEN-induced HCC rats model	In vivo	Anti-inflammatory, antiangiogenic, antiproliferative, and apoptotic effects	^686^
Hippo pathway	TAZ((WWTR1)	Statins(fluvastatin, simvastatin)	HLF and HuH1 cell lines	In vitro	Inhibit cell proliferation	^400^
	MST1, YAP	Salvianolic acid B (SalB)	HepG2, DEN/CCl4/C2H5OH-induced HCC model	In vitro and in vivo	Inhibit cell proliferation and migration, tumorigenesis in vivo	^401^
	Tankyrase	G007-LK	c-Met/sgAxin1 HCC mouse model	In vivo	Synergizes with cabozantinib leading to tumor regression	^390^
	LATS	NIBR-LTSi	Mice with extended hepatectomy	In vivo	Accelerates liver regeneration	^403^
Notch pathway	γ-Secretase	Ginsenoside (Rg3)	HepG2, Huh7 and Huh7 xenograft mouse	In vitro and in vivo	Synergistic antitumor with sorafenib via AKT	^437^
	ADAM17	ZLDI-8	HepG2, Hep3B, Hep3B xenograft mouse	In vitro and in vivo	Synergistic antitumor with sorafenib	^438^
	Notch2	Antagonistic antibody	AKT/Ras HTV mice model	In vivo	Decreased tumor burden	^426^
Epigenetics	Pan-HDAC	Panobinostat	HepG2, Hep3B, HepG2 xenografts	In vitro and in vivo	Delayed growth and prolonged survival	^687^
	Pan-HDAC	Panobinostat	HCC xenografts	In vivo	Combination with sorafenib decreased tumor volume and increased surviva	^688^
	Pan-HDAC	Suberanilohydroxamic A cid (SAHA)	LCL-PI 11	In vitro	Growth inhibition and apoptotic induction	^689^
	Pan-HDAC	HL23	PLC/PRF/5, cMHCC97L orthotopic HCC mouse model	In vitro and in vivo	Suppressed HCC progression and metastasis	^690^
	Pan-HDAC	MPT0G009	Hep3B cell lines and xenograft models	In vitro and in vivo	Induced apoptosis and growth arrest	^691^
	Pan-HDAC	AR-42	Huh7 cell lines and xenograft models	In vitro and in vivo	Radiosensitize in inhibition of HCC cell growth	^692^
	Pan-HDAC	Belinostat	Subcutaneous Hepa129 murine HCC model	In vivo	Enhanced anti-tumor efficacy of checkpoint inhibitors	^464^
	Pan-HDAC	Quisinostat	HCCLM3 xenograft model	In vitro and in vivo	Synergetic with sorafenib, facilitated G0 G1 cycle arrest and apoptosis	^693^
	HDAC1	Trichostatin A	Huh7, HLE, HLF, HepG2, HepG2 xenografts	In vitro and in vivo	Inhibit cell proliferation, induce apoptosis Sensitize HCC cells to enhanced NK-cell mediated killing	^694,695^
	HDAC-3,6,8	Droxinostat	SMMC-7721 and HepG2	In vitro	Induce apoptosis	^696^
	HDAC8	PCI-34051	Hepa1-6 orthotopic HCC mouse model	In vivo	Elevated intratumoral CD8+ T cell infiltration, reduced tumorigenicity, d elevated intratumoral CD8+ T cell infiltration, reduced tumorigenicity alone or combined wth anti-PDL1	ii^473^
	Class I and class II HDAC	Valproic Acid	HepG2, Huh7	In vitro	TRAIL-induced apoptosis and chemotherapy sentization	^697,698^
	Class I, class IIa and IV HDAC	Resminostat	Huh7, HCC-LM3, SK-Hep-1, Huh7 xenografts, HepG2, Hep3B, SMMC-7721, patient-derived primary HCC cells, DEN-induced HCC mouse mode	In vitro and in vivo	Activate apoptosis	^699–701^
	BRD	JQ-1	HepG2, N-Ras/c-Myc HTVi HCC mouse model; Huh7, Hep3B and Hepa129;DEN/CDAHFD diet-induced mouse model of NASH-driven HCC	In vitro and in vivo	Synergetic with anti-PD1 antibody; Reduce tumor aggressiveness	^474–476^
	BRD4	i-BET762	Hepa1-6 orthotopic HCC mouse model	In vivo	Combined treatment with anti-PD-L1 synergistically enhanced TILs, resulting in tumor eradication and prolonged survival in the fibrotic-HCC mouse model.	^477^
	DNMT	Guadecitabine (SGI-110)	SNU398, HepG2 and SNU475	In vitro	Anti-proliferation effects	^702^
	DNMT	5-Aza-CdR (DAC)	SNU5, HepG126, and SNU2	In vitro	Synergistic anti-tumor effect with EZH2 inhibitor (GSK1 26)	^472^
	G9a and DNMT1	CM-272	HepG2, PLC/PRF-LX2 mixed tumors	In vitro and in vivo	Inhibit cell proliferation and tumor growth	^469^
	G9a (EHMT2)	UNC0638, BIX01294	BEL7402, SMMC-7721	In vitro	Inhibit cell growth	^470^
	EZH2	GSK343	Hepa1-6 subcutaneous xenograft mode	In vivo	Tumor suppression	^468^
P53 signaling	wildtype p53	p53 mRNA delivery by nanoparticles	Hep3B cell lines and xenograft models, p53-null orthotopic and ectopic models of murine HCC	In vitro and in vivo	Tumor suppression, synergetic with anti-PD1 antibody	^495,496^
	wildtype p53	Adiponectin	HepG2	In vitro	Suppress tumor through apoptosis	^497^
	mutp53-Y220C	SLMP53-2	Huh7 cell lines and xenograft models	In vitro and in vivo	Growth inhibition, synergistic effect with sorafenib	^498^
	p53-bax	Oleanolic acid	HepG2 cell lines and wildtype mice	In vitro and in vivo	Enhanced apoptosis and reduced hepatotoxicity, synergistic effect with cisplatin	^499^
	p53-bax	Acetylshikonin	SMMC-7721 cell lines and H22 mouse model	In vitro and in vivo	Growth inhibition, enhanced apoptosis	^500^
	Bcl-2	ABT-199	Huh7, Hep3B, HepG2 cell lines	In vitro	Induce cell death in combination with MIK665	^505^
	Bcl-2	Obatoclax	HepG2 and Hepa1-6 cell lines, Hepa1-6 and Hepa1c1c7 xenograft models	In vitro and in vivo	Tumor suppression, synergetic with anti-PD1 antibody	^504^
	Fas/FasL	Scutellarein	Hep3B cell lines	In vitro	Growth inhibition, induce apoptosis and cell cycle arrest	^506^
	Caspase 8	Garciniaxanthone I	HepG2 cell lines	In vitro	Induce apoptosis and inhibit migration	^507^
	Cyt-C	Compound 19b	Huh7, Hep3B, HepG2 cell lines	In vitro	Induce apoptosis	^508^
	Cyt-C	Lycorine	HepG2 cell lines and xenograft models	In vitro and in vivo	Induced apoptosis	^509^
	CDK4/6	Palbociclib(PD-0332991)	PLC/PRF/5, HepG2, Hep3B	In vitro	Growth inhibition	^513^
	CDK4/6	Abemaciclib, ribociclib	HUH7, SNU398, HepG2	In vitro	Simultaneous combination of abemaciclib with lenvatinib reduced 3D cell growth, and impaired colony formation and cell migration	^514^

### Immune-related signaling pathways

Immune-related signaling pathways refer to the intricate regulatory networks that govern the dynamic interplay between the immune system and malignant cells. These pathways can influence the immune response against HCC, thereby affecting tumor growth, progression, and the effectiveness of treatment.

### Cellular signaling pathways

Mutations or activation of pathways such as CTNNB1/WNT-β-catenin, TGF-β, MYC, TP53, ARID1A, and CDK20 exert profound effects on immune responses and immune cell recruitment.^4,515^ For example, CTNNB1 mutations diminish CCL5 expression, impairing dendritic cell (DC) recruitment, and reducing NKG2D ligand expression, thus hindering natural killer (NK) cell-mediated responses.^516,517^ About a quarter of HCC harbor β-catenin mutations, correlating with reduced lymphocyte infiltration and potential resistance to PD-1 blockade, although clinical validation is needed. TP53 mutations occur in ~40% of HCCs overall but are enriched in non-inflamed HCCs, and loss of p53 function promotes the recruitment of immunosuppressive cell.^11,518,519^ while ARID1A mutations have a dual effect on antitumor immunity by affecting mismatch repair and IFNγ signaling.^520^ Genetic alterations in PTEN, RAS, and LBK1 result in lymphocyte depletion and exclusion phenotypes. MYC overexpression upregulates PD-L1, and CDK20 activation recruits myeloid-derived suppressor cells (MDSCs),^521^ suppressing T cell activity. Chromosomal gains at 6p21, containing VEGFA, lead to overexpression of immunosuppressive cytokines.^522,523^ STAT3 activation leads to the production of cytokines like TGF-β, interleukin-17 (IL-17), and VEGF.^524^ Additionally, STAT3 activation can inhibit the immune response orchestrated by T helper type 1 cells (Th1) and further contribute to ICI resistance.^525^ These effects collectively promote an immunosuppressive tumor microenvironment.

### ICIs

ICIs are regulators of the immune system. These pathways are indispensable for self-tolerance, which prevents the immune system from attacking cells indiscriminately. Inhibitory checkpoint molecules are targets for cancer immunotherapy due to their potential for use in multiple types of cancers.

Currently approved checkpoint inhibitors encompasses the blockade of CTLA-4, PD-1 and PD-L1. while additional immune checkpoints in HCC, such as TIM-3 (T cell immunoglobulin domain and mucin domain-3), LAG-3 (Lymphocyte activation gene protein-3) and TIGIT (T cell immunoglobulin and ITIM domains), will also be briefly discussed hereafter.

### PD-1/PD-L1

The immune checkpoint molecule, belonging to the CD28 family, dampens T cell activity during the immune response and prevents autoimmune injury by binding to its ligands PD-L1 or PD-L2. This interaction inhibits the stimulation signal of the T cell receptor (TCR).^526^ PD-1 is expressed on various immune cells, including activated T cells, B cells, NK cells, and dendritic cells (DCs).^527–529^ The expression of PD-L1 can be observed in both tumor cells and antigen-presenting cells (APCs), whereas PD-L2 is predominantly expressed on DCs and macrophages.^530,531^ The expression levels and impacts of PD-L1 and PD-L2 on immune responses are highly context-dependent and can vary significantly across different tissues and disease states.^532^ The expression of PD-L1 on TAMs can attenuate the anti-cancer immune response through its interaction with PD-1 on CD8+ and CD4+ T cells.^533^ The full comprehensive of the significance behind PD-L1/PD-1 expression in immune cells remains elusive.

PD-1 attenuate positive signals from TCR and CD28, affecting downstream pathways like PI3K-AKT, RAS, and ERK.^534^ Additionally, PD-1 inhibits T cell function by upregulating transcription factors such as BATF (Basic Leucine Zipper ATF-Like Transcription Factor), and modulating metabolic pathways to reduce glycolysis while promoting lipid degradation and β-oxidation.^535^ This leads to decreased cytokine Secretion, thereby aiding cancer cells in evading immune responses.^536^ PD-1/PD-L1 expression in Tregs exacerbates immune suppression and exhaustion in the tumor microenvironment, influencing Treg differentiation, maintenance, and function.^536^

It’s important to note that while PD-1/PD-L1 axis inhibition primarily affects T cells, the mechanism may differ in B cells. In B cells, PD-1 activation recruits SHP-2 to dephosphorylate BCR pathway molecules, inhibiting PI3K, ERK, and PLCγ2 pathways, disrupting calcium signaling and inhibiting B cell growth.^537^ PD-1 overexpression in B cells induces T cell dysfunction through an IL-10-dependent pathway, promoting tumor progression. PD-1 + B cells inhibit T cell expansion and viability, with PD-L1 blockade enhancing T cell proliferation and viability.^538^ HDAC6-depleted T cells stimulated PD-1/PD-L1 expression and synergistically sensitized advanced HCC to ICIs, suggesting potential for HCC immunotherapy.

The specific microenvironment created by tumor-releasing factors, LPS and hypoxic conditions can induce the expression of PD-L1 in MDSCs.^539,540^ The immunosuppressive effects of MDSCs on T cells activated by anti-CD3 and anti-CD28 are mediated through the binding of PD-1 on T cells and PD-L1 on MDSCs, leading to T cell inhibition. Additionally, MDSCs can activate the PI3K/AKT/NF-kB pathway in B cells via the PD-1/PD-L1 axis, inducing a subset of immunosuppressive regulatory B cells (Bregs) characterized by the absence of PD-1 and the presence of PD-L1.^541^

Overall, the PD-1/PD-L1 axis is indeed a key player in shaping the immunosuppressive microenvironment of HCC.^542^ The removal of immunosuppression by PD-1/PD-L1 inhibitors is believed to enhance the killing effect mediated by antitumor T cells, while also promoting T cell proliferation and infiltration into the tumor microenvironment (TME) for inducing an antitumor response.^543^ Additionally, it can enhance CD8+ T cell activation in tumor-draining lymph nodes (TDLN) and rejuvenating dysfunctional CD8+ T cells within the tumor.^543^ The anti-PD-L1 antibody, by activating the mTOR pathway in T cells, enhances the immune response not only through T cells but also indirectly affects macrophages. Activated T cells can promote macrophage activation, leading to their transformation into inflammatory and proliferative types that can contribute to a more effective anti-tumor response, which has potential as an alternative cancer treatment.^544,545^

### Inhibitors of PD-1/PD-L1

In the advanced liver cancer field, there are currently FDA-approved PD-1/PD-L1 inhibitors, including nivolumab (PD-1 inhibitor), pembrolizumab (PD-1 inhibitor), camrelizumab (PD-1 inhibitor), tislelizumab (PD-1 inhibitor), durvalumab (PD-L1 inhibitor), atezolizumab (PD-L1 inhibitor) and durvalumab (PD-L1 inhibitor). Nivolumab is the first anti-PD-1 drug used for HCC, showing promising efficacy and safety in the CheckMate 040 trial, a global phase III clinical study. It demonstrates good outcomes among patients with advanced liver cancer, regardless of whether they received prior sorafenib treatment, without triggering viral outbreaks in patients with HCV or HBV hepatitis.^546^ Compared to sorafenib, nivolumab fail to enhance OS but demonstrated remarkable clinical efficacy, exceptional safety profile, and a notably higher rate of complete response, thereby improving patients’ quality of life.^547^ However, for individuals unable to use TKI or antiangiogenesis drugs, especially those at significant risk, nivolumab remains a viable treatment option. Ongoing trials, such as monotherapy in checkmate-9dx (NCT03383458) and combinations with ipilimumab (NCT01658878), continue to assess nivolumab’s potential as adjuvant therapy and in combination treatments for HCC.

In cohort 1 of the Keynote-224 clinical trial, another PD-1 inhibitor, Pembrolizumab, initially demonstrated efficacy and tolerability in patients with advanced HCC who had previously received sorafenib treatment, leading to FDA approval.^548^ Keynote-394 represents a milestone as the first and only phase III trial globally to achieve positive results using PD-1 inhibitor monotherapy for advanced HCC, providing a new treatment option for HCC.

Camrelizumab (SHR-1210), another human (immunoglobulin) IgG4 monoclonal antibody targeting PD-1, was evaluated in a multicenter, open, randomized phase 2 trial in China.^549^ Among 217 patients treated with camrelizumab, the objective response rate (ORR) was 14.7%, with a median progression-free survival (PFS) of 2.1 months and a median OS (mOS) of 13.8 months. The OS rate at 6 months was 74.4% (3%).

The monoclonal antibody Atezolizumab, targeting PD-L1, has demonstrated remarkable efficacy in the phase III imbrave150 study. When combined with bevacizumab, it exhibited a staggering 56% reduction in the risk of mortality (OS) and an impressive 40% decrease in the risk of disease progression or death (PFS), surpassing sorafenib’s performance. Furthermore, this combination therapy of Atezolizumab and bevacizumab was found to be exceptionally well-tolerated with manageable toxicity.^30^

Importantly, Durvalumab, a humanized IgG1 monoclonal antibody targeting PD-L1, has been recommanded as the first-line treatment for HCC in the 2022 edition of NCCN guidelines. This recommendation is based on its preliminary efficacy observed in patients with non-resectable HCC^550^ Specifically, the Himalaya Phase III study demonstrated that both durvalumab monotherapy and its combination with tremelimumab (D + T) significantly improved OS compared to sorafenib. This evidence supports the inclusion of durvalumab in treatment regimens for HCC, highlighting its potential to offer significant clinical benefits.^551^

### CTLA-4

In the tumor microenvironment, CTLA-4 is predominantly expressed in Treg cells, with some effector T cells also expressing CTLA-4. Thus, CTLA-4 not only directly induces T cell function downregulation but also contributes to Treg-mediated suppression of anti-tumor immune responses.^552^ Treg cells have complex functions including direct suppression of DC cell function, inhibition of effector T cells, secretion of TGF-β, and competition for IL-2 and other cytokines through CD25. Tregs are central inhibitory cells in the tumor microenvironment and exhibit high CTLA-4 expression.^553^ Therefore, by blocking the checkpoint CTL4-4, CTLA-4 inhibitors managed to repair the collapsed immune surveillance system.

The CTLA-4 antibody, Ipilimumab, binds to CTLA-4 on effector T cells, thereby alleviating inhibition and exerting its ADCC function to eliminate Tregs. Tremelimumab was the first CTLA-4 inhibitor used for HCC treatment.^554^

Tremelimumab is a fully human IgG2 monoclonal antibody that blocks the interaction of CTLA-4 with its ligands CD80 and CD86. The concurrent utilization of PD-1 and CTLA-4 may potentially yield synergistic alleviation of inhibition and complementary elimination

The initial concerns regarding the potential for ICIs to trigger viral outbreaks in HCC patients with HCV or HBV were addressed in the first pivotal trials, which unequivocally demonstrated the safety of ICIs in these populations. In a groundbreaking move, researchers evaluated the antiviral activity of a CTLA-4 blocker in HCV-infected patients and observed no cases of fulminant hepatitis, establishing the favorable safety profile of ICIs.^555^ Similarly, in the Checkmate-040 trial’s phase 1/2 dose escalation and expansion cohort, no patients with HBV infection experienced hepatitis attacks. This marked the first inclusion of chronic HBV patients in clinical trials of immune checkpoint blockers, setting the stage for subsequent efficacy assessments of immunotherapy in HCC.^556^

### TIM-3/LAG-3/TIGIT

TIM-3, also known as hepatitis A virus cellular receptor 2 (HAVCR2), is an immune inhibitory surface molecule expressed on various immune cells, including T cells, Treg cells, DCs, NK cells, macrophages, and cancer cells. Activation of TIM-3 leads to immune exhaustion of CD8+ T cells and can induce macrophage polarization towards the M2 type, thereby promoting tumor growth through increased secretion of interleukin-6 (IL-6).

LAG-3 is another immune checkpoint that inhibits T cell function and is expressed on tumor-infiltrating lymphocytes (CD4+ and CD8+ T cells), Treg cells, NK/T cells, B cells, NK cells, plasmacytoid DC (pDCs), and TAMs. The expression of TIM-3 and LAG-3 are associated with a poor prognosis in human cancers. TIGIT is expressed on activated NK and T cells, as well as on Treg and Th cells under resting conditions.

Several TIM-3 and LAG-3 agents are currently in clinical development, TIM3 monoclonal antibody TSR-022 (NCT03680508) and with a phase II study combined with anti-PD-1 antibody in HCC patients and mainly include INCAGN02385 (NCT03538028, monotherapy), Relatlimab (NCT04567615) and LAG-3 monoclonal antibody XmAb22841 (NCT03849469 combined with PD-1. At ESMO 2022, LAG-3 monotherapy INCAGN02385reported good tolerability for further investigation in phase Ib/II studies (NCT04370704, NCT05287113) in combination with other immunotherapies. TIGIT antibody Tiragolumab with atezolizumab and bevacizumab showed 2.83-fold higher objective response rate compared to the control therapy.

### Gut microbiota

The human gastrointestinal tract harbors over 100 trillion microorganisms including bacteria, fungi, viruses and archaea that make up the gut microbiota. The gut microbiota carries out critical functions for its host.^557^ As the first organ to encounter enterally absorbed nutrients and microbial metabolites, the liver and the gut microbiome interacted on bile acids conversion and metabolism.^558^

The intricate relationship between gut microbiota and HCC is increasingly recognized, with various studies highlighting its role in both HCC development and treatment response. Microbiota profiles have been linked to clinical-pathological characteristics of HCC patients, such as AFP, ALT, and AST levels, and have shown predictive value for HBV-related HCC microvascular invasion.^559,560^ Additionally, the presence of specific gut microbes, such as B. longum, has been associated with improved liver function recovery in HCC patients during the perioperative period.^561^

Moreover, aberrations in gut microbial-derived metabolite signaling pathways, including toll-like receptor (TLR) and farnesoid X receptor (FXR) signaling, have been implicated in hepatocarcinogenesis.^562–564^ Gut microbiota also serve a role in regulating hepatic NKT cells and anti-tumor immunity, affecting both primary and metastatic liver tumors.^565–567^ Gut microbiota profiles holds promise as non-invasive biomarkers to predict treatment response and guiding therapeutic interventions in HCC patients.^568^

The modulation of gut microbiota is feasible through diet, probiotics, prebiotics, and antibiotics may affect the therapeutic effect of ICIs.^569^ Preclinically, depletion of the microbiota by gut sterilization or antibiotics administration protected against HCC development upon HCC animal models_._^517,562,566,570,571^ Lactobacillus brevis and B. pseudolongum are promising probiotic for metabolism-related HCC prevention.^572,573^ Except above potential treatment, administration of atorvastatin, a cholesterol-lowering drug, and gut microbiota manipulation may also be effective strategies for NAFLD– HCC prevention.^574^ Furthermore, gut microbiota composition or their metabolites has been linked to treatment response and outcomes in HCC patients undergoing immunotherapy, such as with nivolumab and pembrolizumab.^575,576^ Antibiotic treatment can modulate the gut microbiota, affecting bile acid metabolism, hepatic inflammation, and anti-tumor immunity, ultimately impacting the development and progression of HCC.^577–579^ Preclinical studies suggest that antibiotic therapy may reduce secondary bile acids associated with hepatic inflammation and metabolic HCC development, which can enhance anti-tumor immunity.^580^

In the context of immunotherapy, conflicting results have been observed with antibiotic administration at the begining of ICI treatment, with some studies reporting decreased efficacy of ICIs while others show longer progression-free survival (NCT02021253). Probiotic BIFICO during the preoperative phase of HCC patients was capable of accelerating postoperative liver function recovery (NCT05178524). Above all, the clinical therapeutic effectiveness of probiotics in HCC patients is undefined and must considering the issue of dose- and time-finding. New strategies fecal microbiota transplantation (FMT) is becoming a novel, direct and more effective approach to restore gut homeostasis and potentially improve ICI efficacy.

### The immune microenvironment and HCC etiology

**Fig. 5:**
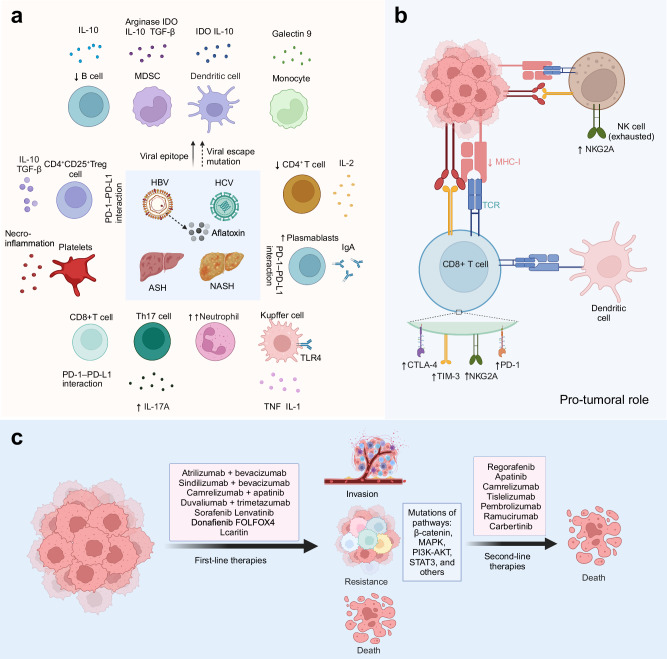
The landscape of immune microenvironment and drug strategies in HCC. **a** The distinct viral and non-viral etiological mechanisms of hepatocellular carcinoma (HCC) are associated with an unique immune suppressive niche. **b** Immune effectors targeting HCC cells. **c** Clinical strategies for HCC. Figure was created with biorender.com

HCC often arises in the setting of chronic liver inflammation, primarily triggered by innate immune activation. HCC caused by different factors may exhibit distinct immune dysfunctions. For instance, chronic viral infections can lead to pro-inflammatory innate immune responses and aberrant adaptive immune reactions, while non-viral HCCs may involve specific subsets of cells and immune cells. Studies suggest a distinctive pro-tumorigenic adaptive immune response in non-viral HCC, characterized by CXCR6+CD8+ T cells with low FOXO1 expression triggering auto-aggression in response to metabolic stimuli in NASH.^581,582^ Therefore, understanding the unique contributions of different HCC causes in shaping the tumor microenvironment is crucial for identifying potential mechanisms that could be targeted for effective immunotherapy strategies (Fig. 5).

### Virus-HCC

Immune responses to HBV and HCV can either promote or inhibit carcinogenesis.^583–586^ Platelets and HBV-specific T cells play dual roles in driving or inhibiting HCC development, respectively. Chronic HBV infection commonly induces a tolerogenic microenvironment in the liver characterized by upregulation PDL-1 and IL-10 in Breg, increasing susceptibility of HBV-specific T cells to apoptosis induced by TIGIT+NKG2D+NK cells, limiting effective anti-tumor immunity.^587^ Highly suppressive PD-1hi Treg cells selectively enrich in HBV-related HCC and correlate with poor prognosis.^588^ In contrast, HCV evades immune surveillance by inducing dysfunctional CD8+ T cells and upregulating immune checkpoint proteins. These cells exhibit reduced production of IFNγ and expression of CD127, impaired proliferation, and increased expression of PD-1^316^. HCV-infected cells can promote the upregulation of TIM3 expression in CD8+ T cells by releasing exosomes, stimulating monocytes to secrete galectin 9.^589^ Additionally, chronic HCV infection may deplete IL-2-producing CD4+ T cells and increase suppressive CD4+CD25+ Treg cells and virus-specific, IL-10-producing CD8+ T cells, which may contribute to hepatocarcinogenesis.^590,591^ Viral escape mutations and HCV core proteins acting as immune evasion proteins, interfering with MHC I-dependent antigen presentation, may also play roles in HCV-related HCC immune evasion.

Metabolic dysregulation also contributes to virus-related HCC pathogenesis. Disruptions in cholesterol homeostasis, driven by high expression of sterol O-acyltransferase 1 (SOAT1), promote tumor cell proliferation and migration in HBV-related HCC.^592^ Targeting these metabolic pathways presents a novel avenue for immunotherapeutic intervention.

In summary, chronic HBV and HCV infections drive hepatocarcinogenesis through complex interactions involving immune dysregulation and metabolic alterations. Understanding these mechanisms is crucial for developing effective preventive and therapeutic strategies for virus-related HCC.

### Aflatoxin-HCC

Aflatoxin, recognized as one of the most potent naturally occurring human hepatocarcinogens, was classified as a “group 1” human carcinogen.^593^

Aflatoxin contamination is frequently observed in improperly stored food such as maize, peanuts, and tree nuts, significantly augmenting the incidence of HCC.^594^ The identification and elimination of these risk factors have substantially reduced its occurrence among young adults, underscoring the significance of aflatoxin control as a preventive measure.

The mutational landscape of Aflatoxin-HCC is characterized by frequent mutations in driver genes, including previously implicated genes such as TP53, RAS, c-fos and ADGRB1.^595–598^ AFB1 causes human HCC by forming a 249Ser mutation in p53. In the early stage of liver cancer, AFB1 induced mutations of the ras oncogene in liver tissues, which mainly occurred in the GG position of codons 12 and 13, most of which were the transversion of G:C to T:A. Aflatoxin can cause the overexpression of c-fos in tree shrew liver tissue, and promote the occurrence and evolution of liver cancer. Among them, ADGRB1 exhibits the highest frequency of mutation in the aflatoxin-associated cohort. Aflatoxin-associated HCCs display a distinctively high mutation rate primarily consisting of C/A mutants, which often leads to a significant increase in the generation of mutation-associated neoantigens (MANAs).^595^ Importantly, this elevated mutational burden is closely correlated with sensitivity to anti-PD-1/PD-L1 therapy.^599^ ICIs hold great potential as an effective therapeutic option for AF-HCCs originating from high-risk regions and the general population.

HBV can increase the risk of HCC in people exposed to AFB1 by 30 times. Exposure to high concentrations can cause acute hepatitis, therefore, chronic exposure can lead to the development of liver cancer. the single most effective way to reduce HCC risk in regions where AFB1 and HBV co-occur is to vaccinate against HBV in order to eliminate the synergistic effect on risk.^600^

### NASH/NAFLD HCC

Non-alcoholic fatty liver disease (NAFLD) is globally prevalent and can progress to NASH, significantly increasing the risk of HCC.^601^ Unlike viral hepatitis, which features organized inflammatory foci, inflammation induced by NAFLD/NASH typically involves scattered inflammatory infiltrates.^582^ Preclinical evidence suggests that NAFLD/NASH-related HCC is characterized by reduced CD4+ T cell activity within tumors, loss of tumor surveillance function by CD8+PD-1+T cells, and pro-tumorigenic functions of NKT cells and Th17 cells. Responses to ICIs are weaker compared to viral HCC. Additionally, CD4+IL-17A+TH17 cells contribute to NAFLD/NASH-induced HCC by promoting neutrophil recruitment and accumulation of fatty acids.^602^ IgA+ plasma cells with high expression of PD-L1 and IL-10 accumulate in NASH, inhibiting CTL activation and promoting HCC. These findings suggest that PD-L1+B cells, possibly a subset of Bregs, exert potent immunosuppressive effects on T cell responses.

Moreover, obesity, commonly associated with metabolic disorders, may reduce the effectiveness of anti-VEGF therapy, although clinical evidence remains complex. Antiplatelet therapy has shown promise in reducing the development of NASH and NASH-related HCC, mediated through platelet-specific glycoprotein Ib-α (GPIbα) and its interactions with platelets and inflammatory monocytes. This is supported by a nationwide cohort study showing long-term use of aspirin is associated with reduced HCC risk.^603^

In summary, understanding the complex interactions between immune cells and hepatocytes in the development of NASH-related HCC is crucial for devising effective prevention and treatment strategies. Targeting platelet-mediated inflammation, immune dysregulation, and metabolic pathways holds promise in combating the occurrence of NASH-induced liver cancer.

### Alcohol HCC

Alcohol intake contributes to up to 30% of the global burden of HCC. Alcohol increases gut permeability, facilitating the entry of immunomodulatory microbiota-derived molecules like LPS into the liver,^604^ where they have the potential to suppress hepatic immune responses, potentially affecting resident macrophages.^605^ Alcoholic steatohepatitis (ASH) is marked by the intrahepatic accumulation of pro-tumorigenic, immunosuppressive granulocytic MDSCs and inhibition of T cell recruitment to the liver and neutrophils in the liver parenchyma.^606^ Cytokines like IL-1 and IL-17 are implicated in the pathogenesis of ASH-related HCC.^607,608^ Thus, The presence of ASH is likely to induce profound alterations in the repertoire and states of immune cells, as well as modifications in the hepatic cytokine milieu, potentially impeding effective adaptive immune responses against HCC.

## The management update of HCC

### Diagnosis

HCC often shows characteristic imaging features, but approximately 10% of tumors, especially those 1-2 cm in size, may lack these hallmarks, posing diagnostic challenges. Diagnosis typically involves examining resected tissue or biopsy samples. Recently, liquid biopsy has gained traction for HCC diagnosis. Components like ct-ncRNA, cfDNA, ctDNA, CTCs, and extracellular vesicles (EVs) are released into body fluids, enabling fluid biopsy.^609–611^ Analytical methods that isolate cells, proteins, nucleic acids, and vesicles from fluids, could aid early diagnosis and monitor HCC progression.^609^ Despite its potential, challenges remain in component separation and sequencing target selection.

Molecular diagnosis is the embodiment of accurate diagnosis in precision medicine. Diagnosis of HCC is further supported by immunohistochemistry for markers glypican-3 (GPC3: NCT05003895, NCT05103631), epithelial cell adhesion molecule (NCT05028933, NCT03013712), MET (NCT01755767), mucin 1 (NCT02587689), MHC1 (NCT05195294), and TERT (NCT05595473), are being incorporated into these clinical trials. The presence of two or more of these markers increases the diagnostic specificity to 100%. The morphology of HCC has been associated with specific molecular alterations. Radiomics can serve as a clinician decision-making tool for constructing HCC diagnosis models, potentially reducing radiologist error rates.^612^ However, clinical application faces challenges like imaging consistency, radiomics standardization, and predominantly retrospective research. Artificial intelligence (AI), particularly deep learning, shows promise in extracting diagnostic, prognostic, and predictive insights from radiological data, achieving over 90% accuracy in classifying lesions with typical features.^613^ Despite potential as clinical decision support, large-scale validation is needed for differential diagnosis of hypervascular liver lesions. Advances in understanding HCC’s molecular mechanisms and technology will likely optimize diagnostic methods and introduce new detection techniques, enhancing early diagnosis and prognosis.

### Therapeutic advances

Advancements in HCC therapy face challenges from complex tumor environments and resistance mechanisms. Innovations like hepatic arterial infusion chemotherapy (HAIC) combined with anti-PD-1 immunotherapy and TKI show promising transformative potential.^614^ The regimens included triple combination therapy (t-CT: lenvatinib, TACE, plus toripalimab) before surgery should be recommended for HCC patients with macrovascular invasion.^615^ Targeted protein degradation (TPD) with techniques such as intramolecular biovalent glues like CC-122 (Avadomide) in clinical trials with nivolumab or sorafenib suggests new treatment avenues.^616^

Antibody-drug conjugates (ADCs) targeting GPC3 and CD24 demonstrate anticancer activity, through mechanisms like antibody-dependent cellular cytotoxicity (ADCC) and complement-dependent cytotoxicity (CDC).^617,618^ CAR-T cell therapy targeting GPC3 shows efficacy, with ongoing enhancements using IL-7 and CCL19 to boost intratumoral activity.^619^ Additionally, CAR-T cells targeting CD147 and CD133 (NCT02541370) are also beneficial targets for the treatment HCC patients.^620,621^ The adoptive transfer of HBV-TCR-T cells into patients with advanced HBV-related HCC was generally well-tolerated and demonstrated a favorable safety profile. Observations of clinical efficacy provide support for the ongoing development and eventual implementation of this treatment strategy in individuals with advanced HBV-associated HCC (NCT03899415).^622^ DC vaccines loaded with neoantigens improve disease-free survival in post-surgery HCC.^623^ Innovative personalized cancer vaccines (e.g., GNOS-PV02) encoding neoantigens and immune-modulating agents demonstrate strong antitumor activity in advanced HCC, highlighting potent T cell responses (NCT04251117).^624^

In conclusion, these emerging therapies — HAIC combined with dual immunotherapy, TPD approaches like intramolecular biovalent glues, ADC development targeting specific markers, and advanced CAR-T cell therapies — are pivotal in expanding HCC treatment options. Continued research and clinical trials are critical to validate and optimize these approaches for improved patient outcomes.

### Prognosis

During the last decade, there has been an obvious improvement in the five-year OS for HCC, from 18% to 22%.^1,625^ This mainly attributed to early detection of HCC, as well as the recent development of systemic drugs such as Lenvatinib and T+A (Tecentriq+Avastin). However, the prognosis of HCC with different etiology varies quite a lot. For exmaple, diagnosis for HCC caused by obesity or non-viral are not currently sensitive enough. Although new detection methods develop, The clinical evidence is limited.

Research indicates that HBV has the most significant impact on OS, followed by HCV, metabolic disorders, and alcoholic liver disease.^626^ A SEER study in 2020 reported median survival after HCC diagnosis as 10.3 months for HBV, 8.3 months for HCV, 7.6 months for metabolic disorders, HBV, 8.3 months for HCV, 7.6 months for metabolic disorders, and 6.1 months for alcohol-related causes. Alcohol and metabolic-related HCCs exhibit higher mortality rates potentially due to delayed diagnosis rather than inherent aggressiveness.^627^ Among liver-related deaths in the United States between 2008 and 2018, there was a substantial decline observed in HCV-related deaths. The prevalence of NAFLD-associated HCC increased from 2.6% in the period of 1995-1999 to 19. 5% during 2010–2014, while the proportion attributed to HCV decreased from 43.6% to 19.5%.^628^ This highlights the effectiveness of HBV vaccination and HCV antiviral therapy in preventing virus-associated liver cancer; however, no strategy currently exists for chemoprevention of non-viral liver diseases.

The prognosis of HCC is gender-dependent, age-dependent and regional-dependent. Across nearly every country, HCC incidence and mortality differ by sex, with rates 1.2–3.6 times higher in men than in women.^629,630^ The mortality rates are higher among elderly patients with HCC. HCC patients in various countries has different outcomes. Treatment choices also influence prognosis of HCC. Lenvatinib has been shown to prolong survival among non-viral HCCs and MAFLD.^631^ While microwave ablation may offer superior long-term recurrence rates when compared to radiofrequency ablation.^632^

### Challenges and directions

There are still several challenges in the field of HCC. Firstly, many of the signaling pathways and targeted inhibitors that have been summarized above exhibit reduced efficacy specifically in HCC compared to other types of cancers, possibly due to the unique characteristics of HCC. Secondly, the absence of clearly defined biomarkers for HCC hinders accurate treatment strategies for patients including targeted therapy or immune therapy,^633–637^ especialliy in ICI. Traditional markers like PD-L1 expression have failed to predict response to nivolumab and pembrolizumab.^547,548,638,639^ Other potential biomarkers, such as high tumor mutational burden (TMB) or microsatellite instability (MSI), are limited in HCC. Tumor heterogeneity and clonal evolution are the underlying mechanisms.^640,641^ Emerging evidence suggests that activated Wnt/ β-catenin signaling is associated with primary resistance to immunotherapy, particularly in ‘cold' tumors lacking T-cell infiltration. Identifying predictive biomarkers are critical due to optimize treatment outcomes in advanced HCC.^642–645^ Furthermore, future clinical applications for treating HCC will likely involve combinations of two or more drugs to overcome the resistance. In additon to the agents, ICI also combined with TACE, radiotherapy and ablation. These have been tested in the clinical phase III. While determining the optimal combination and sequence of FDA-approved or investigational ICIs, as well as other treatment modalities are difficult. Specifically, the classical approach to first surgically remove resectable lesions and/or regional lymph nodes, followed by postoperative (adjuvant) therapy, is gradually giving room to treatment schedules in which neoadjuvant (chemo) immunotherapy is administered before surgery. However, there is a lack of clarity regarding the optimal combination methods, administration sequence, and timing; hence exploring new adjuvant treatment options over the next few years becomes crucial to enhance patient response rates. Lastly, With the rapid development of cellular immune technology and genetic engineering technology, application of adoptive cell therapy, CART, oncolytic virus and vaccine in HCC will benefit more HCC patients. Recent advances related to cancer genomics, proteomics, systems biology and AI suggest new perspectives in precision medicine.

## Conclusion

This review provides valuable insights into the treatment landscape of HCC and its potential application across different cancer types and pathological subtypes. Novel treatment regimens have shown promise in improving survival rates and reducing severe toxicities in HCC patients. Ongoing studies hold the potential to refine therapeutic strategies and identify predictive markers for favorable outcomes. While small molecular inhibitors remain in high demand due to their moderate adverse effects, further efforts are needed to enhance treatment response. Comprehensive research efforts continue to expand our understanding of HCC mechanisms, offering potential avenues for prevention and treatment. The clinical translation of new inhibitors remains critical, and combination therapies involving novel agents and traditional regimens hold significant promise in advancing HCC treatment outcomes.
